# NMR Metabolomics and DNA Sequencing of *Escherichia coli* and *Staphylococcus aureus* Cultures Treated with Hydrolyzable Tannins

**DOI:** 10.3390/metabo13030320

**Published:** 2023-02-21

**Authors:** Valtteri Virtanen, Elina Puljula, Gemma Walton, Martin J. Woodward, Maarit Karonen

**Affiliations:** 1 Natural Chemistry Research Group, Department of Chemistry, University of Turku, FI-20014 Turku, Finland; 2 Department of Food and Nutritional Studies, The University of Reading, Reading RG6 6AH, UK; 3 Folium Science, Science Creates, Midland Road, Old Market, Bristol BS2 0JZ, UK

**Keywords:** antibacterial, bioactive metabolites, in vitro biological activities, metabolome profiling, polyphenols, tannin, untargeted metabolomics

## Abstract

*Escherichia coli* and *Staphylococcus aureus* are globally among the most prominent bacterial strains associated with antibacterial resistance-caused deaths. Naturally occurring polyphenols, such as hydrolyzable tannins, have been shown to potently inhibit *E. coli* and *S. aureus*. The current study investigated the metabolome changes of *E. coli* and *S. aureus* cultures after treatments with different hydrolyzable tannins using an NMR metabolomics approach. Additionally, the effect of these tannin treatments influencing a more complex bacterial system was studied in a biomimetic setting with fecal samples inoculated into the growth medium. Metabolite concentration changes were observed in all three scenarios: *E. coli*, *S. aureus*, and fecal batch culture. The metabolome of *E. coli* was more altered by the tannin treatments than *S. aureus* when compared to control cultures. A dimeric hydrolyzable tannin, rugosin D, was found to be the most effective of the studied compounds in influencing bacterial metabolome changes and in inhibiting *E. coli* and *S. aureus* growth. It was also observed that the tannin structure should have both hydrophobic and hydrophilic regions to efficiently influence *E. coli* and *S. aureus* growth.

## 1. Introduction

Antibacterial resistance is a globally growing concern that has increasing impacts on our everyday life [1]. It is a complex issue because there is an ever-growing number of human-ailing bacterial strains that have varying amounts of resistance to commonly used antibacterial medication [2]. Moreover, food-producing animals and agriculture also suffer from bacterial strains with increased resistance, often the result of using antibacterial agents as growth promoters, which in turn affects their productivity and can also be a source of infection for humans [3]. Both of these issues have substantial economic impacts in the form of requiring larger treatment dosages, which only expedites the underlying problem, or forcing consumers to use other more costly and often prolonged treatments [2]. Alternative solutions are required to alleviate this problem more effectively. Natural products such as hydrolyzable tannins (HTs) are one option that have shown very promising anti-inflammatory [4,5], antiviral [6,7,8,9], and antibacterial properties [10,11,12,13,14]. HTs are a structurally varying group of complex compounds that have many favorable bioactivities, such as antioxidant activity [15,16], protein precipitation capacity [17,18,19,20,21], and other interesting macromolecule interactions [22,23]. Structural features and physicochemical properties such as hydrophobicity have been linked to their antimicrobial potential [24], but more information is needed about the mechanisms behind these beneficial interactions to better utilize them in future applications.

Untargeted metabolomics has become a staple method to study the changes in many different sample environments due to its relative ease of use and because it requires little to no previous knowledge about the studied metabolites. Untargeted metabolomics with nuclear magnetic resonance (NMR) spectroscopy is highly reproducible and can quantitatively measure all proton-containing compounds in a solution [25,26]. NMR analyses also benefit from having very few sample preparation steps, thus ensuring minimal sample modification before analysis. Metabolomics with NMR is effective to the micromolar level, and while not as sensitive as mass spectrometry (MS)-based metabolomics, it is still very effective, and the other advantages often outweigh the lower sensitivity. Metabolomic datasets are typically very large with high dimensionality, which is why powerful tools such as multivariate analysis with principal component analysis (PCA) are often used to visualize them to draw meaningful conclusions.

**Figure 1:**
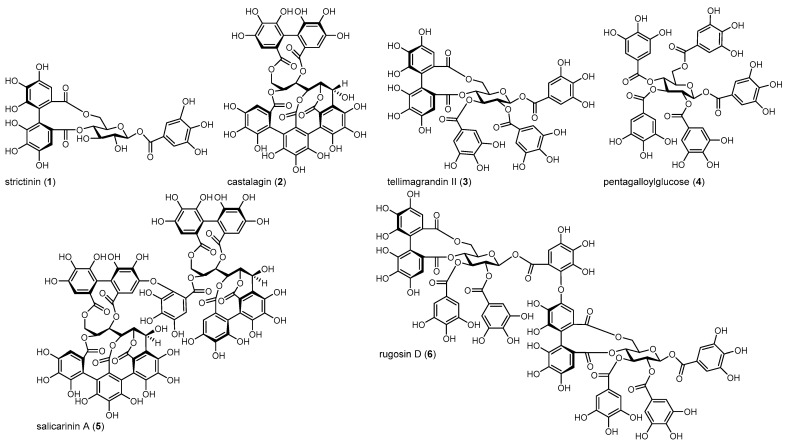
Structures of hydrolyzable tannins used in this study: **1**, strictinin; **2**, castalagin; **3**, tellimagrandin II; **4**, pentagalloylglucose; **5**, salicarinin A; and **6**, rugosin D.

In this study, we applied untargeted biofluid NMR metabolomics to study the changes that different hydrolyzable tannin (HT) treatments caused in the cultures of *Escherichia coli* and *Staphylococcus aureus* at different growth times. In addition to these strictly bacterial growth experiments, a more biomimetic fermentation study was conducted with fecal samples included in the growth medium of *S. aureus* to evaluate the effect of the HTs in this more complex environment. A structurally varying set of HTs (Figure 1) was selected to observe how different structural features can change the metabolome of the studied cultures. Notable structural features of HTs included the number of free galloyl, hexahydroxydiphenoyl (HHDP), and nonahydroxytriphenoyl (NHTP) groups and the conformation of the central cyclic polyol. All of the selected HTs had antimicrobial potential according to previous studies [13,27,28,29,30,31]. In addition, the impact of these HT treatments on the fecal microbial community was assessed using DNA sequencing during the fermentations. 

## 2. Materials and Methods

### 2.1. Reagents

Deuterium oxide (D_2_O) and 3-(trimethylsilyl)propionic-2,2,3,3-*d*_4_ acid sodium salt (TSP) were purchased from Eurisotop, a subsidiary of Cambridge Isotope Laboratories, Inc. (Tewksbury, MA, USA). All other reagents were purchased from Sigma-Aldrich (Seelze, Germany). DNeasy PowerSoil Kit was purchased from QIAGEN Ltd (Venlo, Netherlands).

### 2.2. Bacterial Culture Conditions

Cultures of *Escherichia coli* (APEC46) and *Staphylococcus aureus* (isolated from ham, National Agricultural Research Foundation, Lycovrissi, Greece) were utilized in this study. 

*E. coli* and *S. aureus* were inoculated into M9 medium and grown at 37 °C overnight. The optical density of the cultures was checked at 600 nm after overnight incubation to estimate the growth. The starting concentrations were plated from this point. An aliquot of 2450 µL of autoclaved M9 medium was added to an autoclaved 5 mL bacterial culture flask. Then, 300 µL of either 1 mM or 0.5 mM solution of one of the studied hydrolyzable tannins (**1**–**4** and **6** in PBS) was added to the flask along with 250 µL of *E. coli* or *S. aureus*. The resulting total culture volume was 3 mL. Three replicates were conducted with both tannin concentrations, resulting in six replicates for all studied tannins. Culture flasks were incubated at 37 °C without shaking. Samples for the time points of 0 h, 5 h, and 24 h were collected from each replicate at their respective times after proper mixing of the culture solution. The sample volume was 550 µL from which a 50 µL aliquot was taken for plating. The remaining 500 µL was centrifuged for 10 min at 11,200× *g*, and 450 µL of the supernatant was frozen and stored at –80 °C for NMR analysis. For each time point, four control samples (growth medium and bacterium without HT treatment) were analyzed in conjunction with the treated samples, and eight quality control (QC) samples were additionally prepared from the M9 medium.

### 2.3. Batch Culture Fermentation

Ampicillin-resistant *S. aureus* was utilized in the fecal batch culture (fecal BC) fermentation study. Three separate fermentation experiments were carried out in small (10 mL) batch culture (BC) fermentation vessels. The BC fermentation vessels were filled with 7 mL of basal nutrient medium (peptone water (2 g/L), yeast extract (2 g/L), NaCl (0.1 g/L), K_2_HPO_4_ (0.04 g/L), KH_2_PO_4_ (0.04 g/L), NaHCO_3_ (2 g/L), MgSO_4_·7H_2_O (0.01 g/L), CaCl_2_·6H_2_O (0.01 g/L), Tween 80 (2 mL/L), hemin (50 mg/L), vitamin K1 (10 mL/L), L-cysteine (0.5 g/L), bile salts (0.5 g/L), and resazurin (1 mg/L) and autoclaved. The vessels were gassed overnight with O_2_-free N_2_ before starting the experiment. During the experiment, magnetic stirring was used and the pH and temperature were controlled (pH 6.8 and T = 37 °C).

Fecal samples were collected on the day of inoculation and kept in an anaerobic container for no more than 2 h prior to inoculation. Three donors were used, one donor for each fermentation experiment. Two donors were female, and one was male. The donors had not used any antibiotics in the previous 6 months. The fecal samples were diluted 1:10 in phosphate-buffered saline (PBS; 0.1 M, pH 7.4) and then homogenized in a stomacher (Steward 400) for 2 min at 240 paddle beats per min.

The following components were added into the culture container: 1 mL of fecal sample (10 vol% in PBS); 150 uL of glucose (20 vol%); 1 mL of *S. aureus* (ampicillin-resistant strain), and 1 mL of one of the studied hydrolyzable tannins (2–6; 3 mM in PBS), positive control (10 vol% fructooligosaccharide (FOS; Orafti P95, Beneo, Tienen, Belgium) in PBS), or negative control (PBS). In addition, seven quality control (QC) samples were prepared from the growth medium.

Time point samples for 0 h, 8 h, 24 h, and 48 h were collected at their respective times. A 1000 µL sample was collected, and a 100 µL aliquot was taken from this sample for plating. The plating of the fecal BC set samples was carried out on plates dosed with ampicillin (0.05 mg/mL) to kill all other possible bacteria originating from the fecal samples except the added ampicillin-resistant *S. aureus*. The remaining 900 µL was centrifuged for 10 min with 11,200× *g*; the supernatant was separated from the sediment and both were stored separately at −80 °C for NMR (supernatant) and for DNA sequencing and measuring DNA quantity (sediment). 

### 2.4. Hydrolyzable Tannin Isolation and Characterization

Extraction, isolation, and characterization of the HTs used in this study (Figure 1) followed our previously outlined methods. Briefly, plant material was extracted with acetone:water (4:1, *v*:*v*), lyophilized, fractionated with Sephadex LH-20 gel chromatography, and finally purified with preparative and semi-preparative HPLC [20,21,32]. All purification steps were monitored with UPLC-DAD–MS. 

The UPLC-DAD–MS measurements of the purified HTs were performed with an Acquity UPLC (Waters Corp., Milford, MA, USA) instrument connected via a heated electrospray ionization (HESI) source to a Q Exactive hybrid quadrupole-Orbitrap mass spectrometer (Thermo Fisher Scientific GmbH, Bremen, Germany). The UPLC consisted of a binary solvent manager, a sample manager, a column oven, and a diode array detector (DAD). The used column was an Acquity BEH Phenyl column (2.1 × 100 mm, 1.7 µm; Waters Corporation, Wexford, Ireland). The mobile phase consisted of acetonitrile (A) and formic acid and water (0.1:99.9, *v*:*v*): 0–0.5 min: 0.1% A; 0.5–5.0 min: 0.1–30% A (linear gradient); 5.0–9.5 min: column wash and stabilization. The mass spectrometer was operated in negative ionization using the following parameters: spray voltage, 3.0 kV, sheath gas (N_2_) and auxiliary gas (N_2_) flow rates, 60 and 20 arbitrary units, respectively; auxiliary gas temperature, 300 °C; capillary temperature, 380 °C. Orbitrap was calibrated with Pierce ESI Negative Ion Calibration Solution (Thermo Fischer Scientific Inc., Waltham, MA, USA). Full scan spectra were collected with a mass range of *m*/*z* 150–2000, a resolution of 70,000, and an automatic gain of 3 × 10^6^. Fragmentation spectra were measured with normalized collision energies of 20, 50, and 80 eV in the higher energy collisional (HCD) cell.

NMR experiments of the purified HTs were performed with a Bruker Avance-III spectrometer operating at 600.16 MHz for ^1^H and 150.90 MHz for ^13^C equipped with a Prodigy TCI (inverted CryoProbe) cooled via liquid nitrogen or a Bruker Avance-III spectrometer operating at 500.08 MHz for ^1^H and 125.76 MHz for ^13^C equipped with a Smartprobe (Fällanden, Switzerland). Instruments were operated under TopSpin 3.5 pl 7. Spectra were recorded using acetone-*d*_6_ in 298.15 K. Recorded experiments included standard ^1^H and ^13^C spectra, COSY, NOESY, multiplicity-edited HSQC, HMBC, and selective 1D-TOCSY. Reported chemical shifts are with respect to the solvent signal at *δ*_H_ = 2.05 ppm. The final product purities and MS and NMR data are presented in Supplementary Materials S1. 

### 2.5. NMR Metabolomics

#### 2.5.1. Sample Preparation

The samples were thawed at room temperature and centrifuged (4 °C, 15,000× *g*, 5 min), and 400 µL of supernatant was mixed with 200 µL of NMR buffer (0.5 M phosphate, 0.6 mM TSP, 3 mM potassium phthalate). The sample was transferred into an NMR tube and stored at 6 °C prior to measurement (Bruker SampleJet, Billerica, MA, USA).

#### 2.5.2. Data Acquisition and Preprocessing

NMR analysis of the samples was performed on the 600.16 MHz NMR instrument described in Section 2.4. All spectra were acquired with 64 scans at 25 °C using a water-suppressed 1D NOESY [33] pulse sequence (Bruker’s pulse program noesygppr1dep), a relaxation delay of 5 s, and a mixing time of 100 ms. During acquisition, the free induction decays (FIDs) were collected with 59,522 data points, an acquisition time of 4.5 s, and a spectral width of 6614 Hz. To ensure identical handling of the samples, spectra were recorded using Bruker’s IconNMR automation software for automated tuning and matching as well as gradient shimming using the topshim tuneb tunea routine for each sample.

After acquisition, the data were processed with NMRProcFlow [34], a platform dedicated for batch processing of NMR data for metabolomics using typical spectral processing steps [35]. An exponential line broadening of 0.3 Hz was applied to all FIDs prior to Fourier transformation. All ^1^H NMR spectra were automatically phased, calibrated internally to TSP (*δ* 0.0 ppm), normalized (constant sum normalization) with the reference signal (TSP), baseline corrected (global correction, high correction), and aligned (least square, *δ* 0.05 relative max shift). Each spectrum was binned using the adaptive intelligent binning method, which attempts to avoid issues such as a bin containing area data from two or more peaks which is a typical problem with the traditional binning methods where the spectra are divided into equal-sized bins [36]. The spectra from the *E. coli* set were divided into 464 bins; *S. aureus* set, into 509 bins; and BC set, into 648 bins. The region of suppressed water (*δ* 4.70–4.90) was excluded from the subsequent data analysis.

#### 2.5.3. Multivariate Analysis

The samples in each culture set (*E. coli*, *S. aureus*, and fecal BC) were grouped according to sample collection time points (0 h, 5 h, and 24 h or 0 h, 8 h, 24 h, and 48 h) and according to the treatment, i.e., control or added HT. Multivariate analysis was performed with either R (ver. 4.2.1) [37] using RStudio (ver. 2022.02.0 Build 443) [38] and package “mdatools (ver. 0.13.0)” [39] or SIMCA (ver. 12.0.1.0). 

#### 2.5.4. Individual Metabolite Responses

Significant individual metabolite signals were observed from 1D spectra of the different sample sets with the help of each set’s PCA model loadings (Table 1). The identities of these metabolites were tentatively characterized with the help of the Human Metabolome Database (HMDB) [40], Biological Magnetic Resonance Data Bank (BMRB) [41], Chenomx database, and literature [42,43,44,45,46]. Integrals of the significant metabolites were converted to concentrations with the response of the known concentration of phthalate (1.0 mM) that was included in the NMR buffer using the following equation:

(1) $$C_{M}=\frac{I_{M}}{I_{S}}\times \frac{N_{S}}{N_{M}}\times C_{S}$$

where C_M_ and C_S_ are the concentrations of the metabolite and standard, respectively; I_M_ and I_S_ are the integral areas of the metabolite and the standard, respectively; and N_M_ and N_S_ are the numbers of protons contributing to these resonances, respectively. The reason for using phthalate instead of the internal standard, TSP, for the quantitations was the unfortunate property of TSP binding to leftover proteins in the sample, leading to inaccurate quantitations.

### 2.6. Bacterial Growth with Plate Measurements

Samples from *E. coli* and *S. aureus* bacterial cultures and from the fecal BC fermentation study were diluted in M9 medium/PBS and plated on LB plates. The plates were incubated at 37 °C overnight, and colonies were counted 5 and 24 h after.

### 2.7. Bacterial Culture DNA Sequencing

The DNA was extracted using DNeasy PowerSoil Kit (100) Cat (No./ID:12888-100) following the manufacturer’s instructions. 16S rRNA gene amplicon-based sequencing was performed by the Animal and Plant Health Agency, Surrey, UK. Aliquots of extracted DNA were amplified with universal primers for the V4 and V5 regions of the 16S rRNA gene (U515F (5′-GTGYCAGCMGCCGCGGTA) and U927R (5′-CCCGYCAATTCMTTTRAGT)) [47]. Forward and reverse fusion primers consisted of the Illumina overhang adapters 5′-TCGTCGGCAGCGTCAGATGTGTATAAGAGACAG and 5′-GTCTCGTGGGCTCGGAGATGTGTAATAAGAGACAG, respectively. Amplification was performed with FastStart HiFi Polymerase (Roche Diagnostics Ltd., Hassocks, UK). Amplicons were purified using 0.8 volumes of Ampure XP magnetic beads (Beckman Coulter, Brea, CA, USA). Samples were tagged with a unique pair of indices and the sequencing primer, using Nextera XT v2 Index kits and 2 × KAPA HiFi HotStart ReadyMix. Index-tagged amplicons were purified using 0.8 volumes of Ampure XP magnetic beads (Beckman Coulter). The concentration of each sample was measured using the fluorescence-based Quantifluor assay (Promega, Madison, WI, USA). Concentrations were normalized before all samples were pooled to be subsequently identified by a unique index combination. Sequencing was performed on an Illumina MiSeq with 2 × 300 base reads according to the manufacturer’s instructions (Illumina, Cambridge, UK).

Obtained sequence reads were processed according to the microbiome-helper pipeline [48]. Paired-end reads were merged based on overlapping ends using PEAR [49] before data were filtered for base-calling quality and amplicon length. The processed sequences were classified using the pick open reference OTU process implemented in QIIME v1.9.1 [50] using the Greengenes 16S rRNA gene database [51]. The resulting distribution of OTUs across the multiple samples was further analyzed using QIIME v1.9.1 to summarize the distributions.

## 3. Results and Discussion

### 3.1. NMR Metabolomics

The time-evolved and HT treatment-altered metabolomes of *E. coli*, *S. aureus*, and fecal BC (ampicillin-resistant *S. aureus*) were studied with NMR metabolomics. The measured data were subjected to multivariate analysis to find group (time point and HT treatment) differences in the different culture sets. Additionally, individual metabolites responsible for the largest variation within sets were identified and changes in their concentrations between time points were observed.

#### 3.1.1. Metabolomic Alterations in *E. coli* Culture 

**Figure 2:**
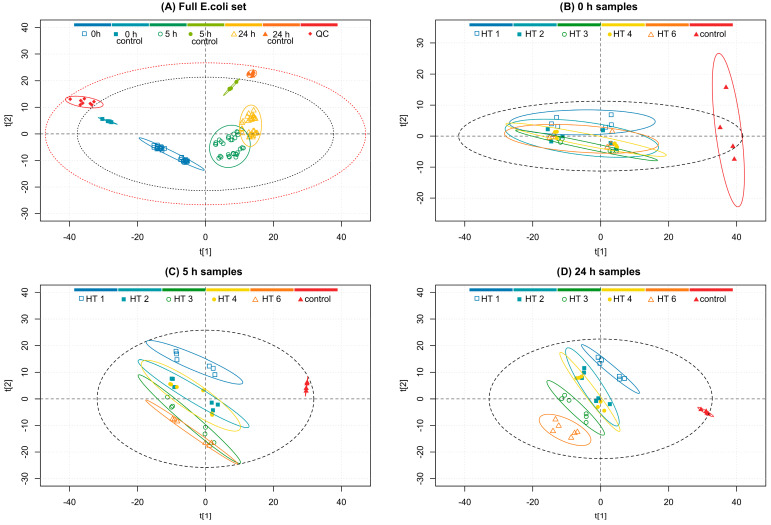
PCA model score plots of the (**A**) full *E. coli* sample set (R^2^X(1) = 0.49, R^2^Y(2) = 0.16), (**B**) samples at the 0 h time point (R^2^X(1) = 0.55, R^2^Y(2) = 0.04), (**C**) samples at the 5 h time point (R^2^X(1) = 0.32, R^2^Y(2) = 0.21), and (**D**) samples at the 24 h time point (R^2^X(1) = 0.34, R^2^Y(2) = 0.16) treated with hydrolyzable tannins (HTs). Plot (**A**) groups according to the time point, time point control, or quality control (QC), and plots (**B**–**D**) groups according to the HT treatment or control. Red and grey ellipses show Hotelling’s T^2^ 99% and 95% confidence intervals, respectively, and group ellipses show 95% confidence intervals. (**A**) 0 h samples (□, dark blue), 0 h controls (■, turquoise), 5 h samples (⚪, dark green), 5 h controls (⚫, light green), 24 h samples (△, yellow), 24 h controls (▲, orange), and quality control samples (♦, red). (**B**–**D**) HT 1, strictinin (□, blue); HT 2, castalagin (■, turquoise); HT 3, tellimagrandin II (⚪, dark green); HT 4, pentagalloylglucose (⚫, yellow); HT 6, rugosin D (△, orange); and controls (▲, red).

The ^1^H NMR data of the *E. coli* set was processed and bucketed as described in Section 2.5.2. Figure 2A displays a PCA plot of the *E. coli* culture sample set with the different time points, their controls, and QC samples colored in different series. The PCA model was constructed with the buckets of the full spectral region of *δ* 0.02–10.10 ppm excluding only the region of suppressed water *δ* 4.7–5.0 ppm. In the PCA plot, the QC samples separated from the actual samples noticeably, as did the control samples of each time point. Additionally, each time point was clustered separately displaying the time-evolved metabolite profile. There was also further clustering within each time point into two separate groups. These inner groups were formed from the two sets using different HT concentrations (three replicates with 0.1 mM HT and three replicates with 0.05 mM HT). This shows that the HT concentration has a considerable effect on the *E. coli* metabolome development.

The effect of the different HT additions to the *E. coli* culture was not visible from the PCA score plot of the full *E. coli* set (data not shown). This was unexpected as it was initially hypothesized that the different HTs would be able to influence the metabolite profile in different ways as they have been reported to inhibit *E. coli* with varying efficiencies [13]. However, the different HT treatments did ultimately reveal differences when samples from specific time points were observed separately. Figure 2B–D display PCA score plots from PCA models on only the specific time point samples and their respective controls. 

Comparing the separation of the group clusters of different HT treatments in Figure 2B–D reveals that the cultures with different HT additions initially seem to cluster together as is expected because the HT additions had not had time to affect the metabolome considerably. However, when the cultivation time increases, the differences between the HT treatments were revealed, and in the plot of 24 h samples (Figure 2D), the group clusters are adequately resolved. The HT treatments were sequenced in the following order based on ascending distance between the HT and control cluster: strictinin (**1**), castalagin (**2**), pentagalloylglucose (**4**), tellimagrandin II (**3**), and rugosin D (**6**). The above order of HT-altered metabolite changes is in line with their previously reported strength of antimicrobial activity against *E. coli* [13]. Moreover, there are grounds for this order based on the different bioactivities of HTs, such as oxidative activity, protein precipitation capacity, hydrophobicity, anthelmintic activity, antimicrobial activity, and lipid interactions as well as the structural features of HTs [12,13,15,20,23,52,53,54,55]. 

The smallest of the studied HTs, strictinin, has a low hydrophobicity [54]. Previously, we have noticed that strictinin does not perturb bilayers of lipid vesicles and does not inhibit the exsheathment of nematode larvae or the growth of bacterial strains such as *E. coli*, *S. aureus*, and *Clostridiales perfringens* effectively [12,13,23]. Additionally, a close structural isomer of strictinin, isostrictinin, which has an HHDP group in the 2,3 position of the glucose core instead of the 4,6 position, has low oxidative activity and low efficacy to inhibit egg hatching or motility of nematodes [15,55]. Although strictinin has not previously shown strong bioactivities, it still altered the *E. coli* metabolome in comparison to the control samples, as seen in Figure 2B–D where its cluster is visibly separate from the controls.

Castalagin and pentagalloylglucose clusters are both close to one another, suggesting that they are both able to modify the *E*. *coli* metabolome in an almost equal way. This observation suggests that the hydrophilic castalagin [56] with an open glucose core and a rigid planar structure due to the intramolecular linkages in the HHDP and NHTP groups [56] has similar effects in some sense to those of the hydrophobic pentagalloylglucose with five flexible galloyl groups [57]. Acyclic structures, such as castalagin, are typically associated with high hydrophilicity [54] and strong oxidative activity [15], whereas the type of structure in pentagalloylglucose is typically associated with high hydrophobicity and strong protein precipitation capacity and was recently shown to strongly interact with lipid vesicles [23,32]. Thus, the structural features, physicochemical properties, and bioactivities of these two HTs are very different, but nevertheless, they alter the metabolome in bacterial strains similarly. Unfortunately, based on the data obtained in this study, more detailed mechanisms or molecular aspects cannot be clarified. 

The phenomenon is further supported by the fact that the samples treated with tellimagrandin II, which has both free galloyl groups and an easily oxidizable HHDP group, clustered further from the control samples than the samples treated with either of the formerly described compounds. This observation might be due to these less hydrophobic structures being unstable in the M9 buffer solution and therefore resulting in common degradation products, such as ellagic acid and valoneic acid dilactone, which are more hydrophobic than the degradation products of the originally more hydrophobic HTs that often produce gallic acid as their main degradation product [58]. The samples treated with rugosin D separated the furthest, suggesting that its structure is the most effective of the studied HTs in changing the metabolome of the studied *E. coli* culture. Rugosin D is a dimeric ellagitannin with multiple free galloyl groups in both of its monomeric units, which as a structural feature is reported to be beneficial in interactions with lipids that are the constituents of bacterial membranes. Additionally, there is an HHDP group in both of the monomeric units acting as the important hydrophilic region of the structure.

**Figure 3:**
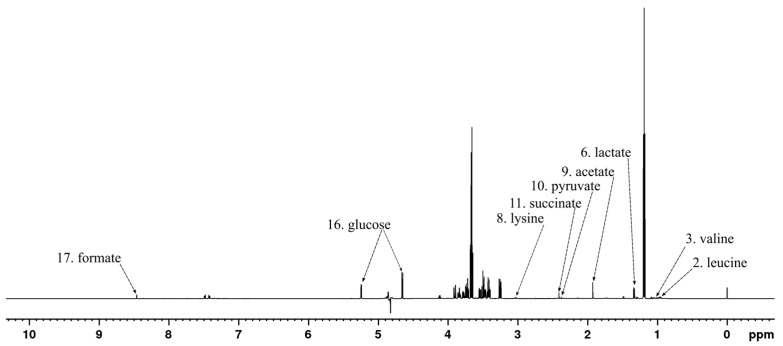
^1^H NMR spectrum of *E*. *coli* culture sample at the 0 h time point after treatment with rugosin D (**6**) with example signals of the significant metabolites labeled according to Table 1.

**Figure A1:**
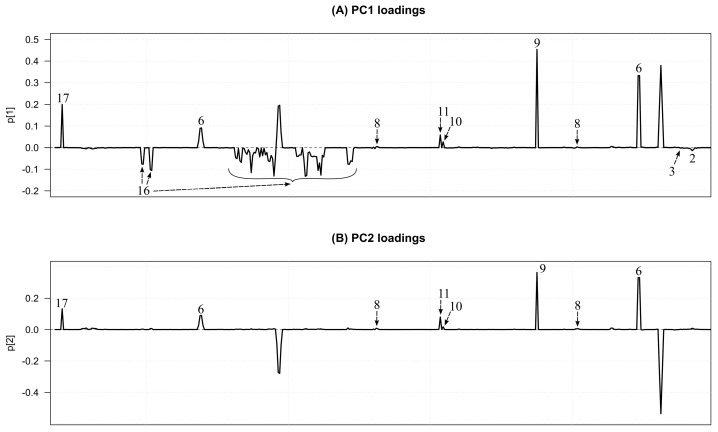
Loading plots of the PCA model of the full *E. coli* sample set: PC1 and PC2 loadings with the x-axis reversed to mimic the chemical shift axis of a ^1^H NMR spectrum. Significant metabolite and their labels are presented in Table 1.

Metabolites that displayed considerable contribution to the principal components of the PCA model shown in Figure 2A were identified based on the associated model loadings (Appendix A Figure A1) and labeled in the ^1^H spectrum in Figure 3. The loading plot shows that most of the negative contribution was attributable to the decreasing amount of glucose that the bacteria are using from the growth medium. Additionally, leucine and valine showed some negative contributions. Lactate, acetate, and formate show significant positive contributions while smaller positive contributions were observed from lysine, pyruvate, and succinate.

**Figure 4:**
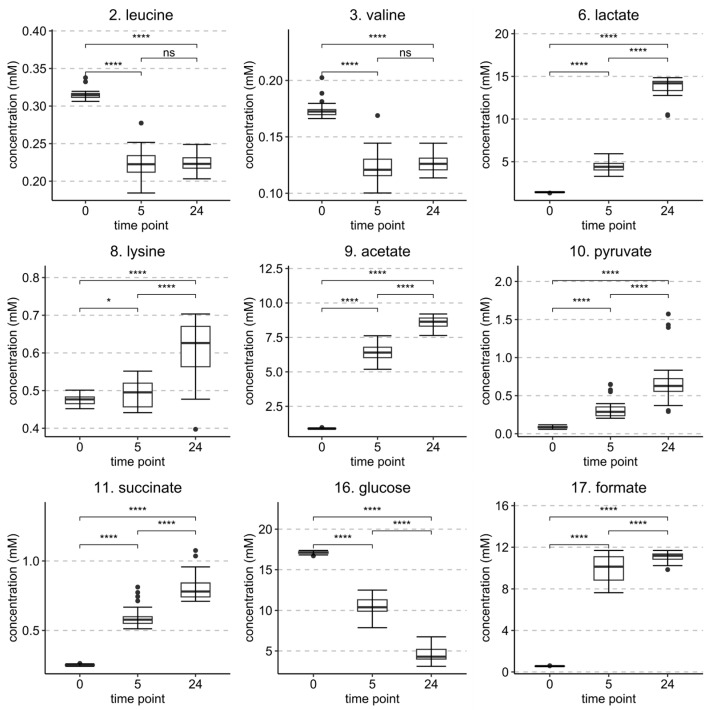
Statistically significant (*p* < 0.05) identified metabolites and their concentrations from the *E. coli* sample set at 0 h, 5 h, and 24 h time points. Metabolites are numbered according to Table 1. *p* values of difference are from a paired *t*-test (*n* = 30). Levels of significance: **** *p* < 0.0001; * *p* < 0.05; ns = non-significant.

Statistically significant changes in the concentrations of metabolites between the time points of 0 h, 5 h, and 24 h are shown in Figure 4 and for the same time intervals but with separated HT treatments in Supplementary Materials Figure S1. A notable decrease in glucose concentration in both monitored time intervals was observed, while leucine and valine decreased significantly only from 0 h to 5 h time points but not significantly from 5 h to 24 h. Lactate, acetate, succinate, and formate concentrations increased in both intervals, which is reasonable as they are produced by microbial fermentation of carbohydrate sources [59]. Lysine and pyruvate levels increased in both time intervals. In the HT treatment-separated plots (Figure S1), differences between the HT treatments were observable for the concentration changes from 0 h to 5 h in the concentrations of lactate, acetate, succinate, and formate (Figure S1). More specifically, the samples treated with tellimagrandin II or rugosin D produced considerably less of these metabolites than samples that were treated with the other HTs, suggesting more efficient inhibition of *E. coli*. The detected order of inhibition efficiency was in line with what was earlier observed directly from the PCA plots, i.e., studied HTs in ascending order: strictinin (**1**), castalagin (**2**), pentagalloylglucose (**4**), tellimagrandin II (**3**), and rugosin D (**6**). 

The increase in the overflow metabolites (acetate, formate, lactate, and succinate) in the growth medium verified that the culture was growing by fermentation [59]. The increases in these metabolite levels and the decrease in glucose also confirmed that the HT treatments were not fully able to inhibit the growth of *E. coli*. However, when compared to the control samples (Figure S1), all HT treatments were able to decrease the rate of growth noticeably. 

#### 3.1.2. Metabolomic Alterations in *S. aureus* Culture

**Figure 5:**
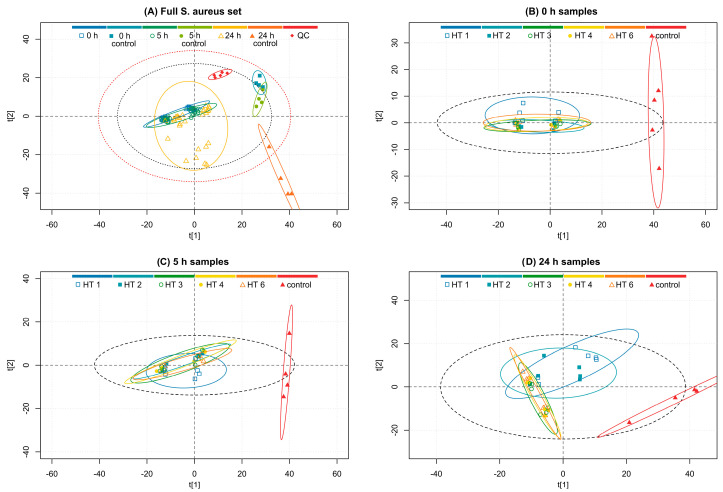
PCA model score plots of the (**A**) full *S. aureus* sample set (R2X(1) = 0.38, R2Y(2) = 0.18), (**B**) samples at the 0 h time point (R2X(1) = 0.58, R2Y(2) = 0.05), (**C**) samples at the 5 h time point (R2X(1) = 0.55, R2Y(2) = 0.20), and (**D**) samples at the 24 h time point (R2X(1) = 0.47, R2Y(2) = 0.19). Plot (**A**) groups according to the time point, time point control, or quality control (QC), and plots (**B**–**D**) groups according to the HT treatment or control. Red and grey ellipses show Hotelling’s T2 99% and 95% confidence intervals, respectively, and group ellipses show 95% confidence intervals. (**A**) 0 h samples (□, dark blue), 0 h controls (■, turquoise), 5 h samples (⚪, dark green), 5 h controls (⚫, light green), 24 h samples (△, yellow), 24 h controls (▲, orange), and quality control samples (♦, red). (**B**–**D**) HT 1, strictinin (□, dark blue); HT 2, castalagin (■, turquoise); HT 3, tellimagrandin II (⚪, dark green); HT 4, pentagalloylglucose (⚫, yellow); HT 6, rugosin D (△, orange); controls (▲, red).

The dataset obtained from the *S. aureus* culture was processed similarly to the previously described dataset for *E. coli* cultures. The PCA score plot constructed from the buckets of the full spectral region of *δ* 0.02–10.10 ppm (excluding only the region of suppressed water *δ* 4.7–5.0 ppm) in Figure 5A shows that the QC samples and time point control samples clustered separately. The time point samples of the *S. aureus* set were initially expected to display similar time-evolved separation as was earlier observed with *E. coli*. However, the actual time point samples were relatively centralized and overlapped with each other considerably in the PCA plot, which was especially true for the samples obtained at 0 h and 5 h. Some kind of separation was observed with the formerly mentioned and the cluster of 24 h samples, but the difference is less evident than that in the case of the *E. coli* set. This lack of time point separation suggested that the metabolome of the *S. aureus* culture was not changing as significantly with time or that the HT treatments are already at the initial 0 h time point changing the metabolome to such an extent that close to no change is observed at the subsequent time points. The reasons behind the similarity of these time points are further discussed in Section 3.2 with the plated bacterial growth results from these time point samples. 

Time-point-specific PCA models (Figure 5B–D) similar to those constructed for *E. coli* were also constructed for *S. aureus*, but they, unfortunately, did not reveal any significant differences between different HT treatments. Only in the final score plot corresponding to the 24 h samples, the clusters of culture samples treated with strictinin and castalagin showed some separation from the clusters of other treatments. These HTs were also found to influence the metabolome of *E. coli* culture the least, so the difference detected here between treatments may be caused by the ineffectiveness of strictinin and castalagin in inhibiting the *S. aureus* culture as well as the more effective HTs. This is supported by the fact that the same HTs have been reported to not inhibit *S. aureus* as effectively as the larger HTs included in this study [13]. Some inner clustering into two groups within each time point was observed due to the two different HT concentrations used but not as remarkably as in the *E. coli* set. 

**Figure 6:**
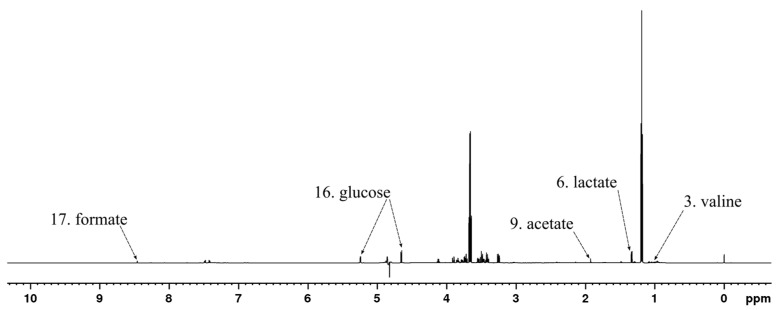
^1^H NMR spectrum of *S. aureus* culture sample at the 0 h time point after treatment with rugosin D (**6**) with example signals of the significant metabolites labeled according to Table 1.

**Figure A2:**
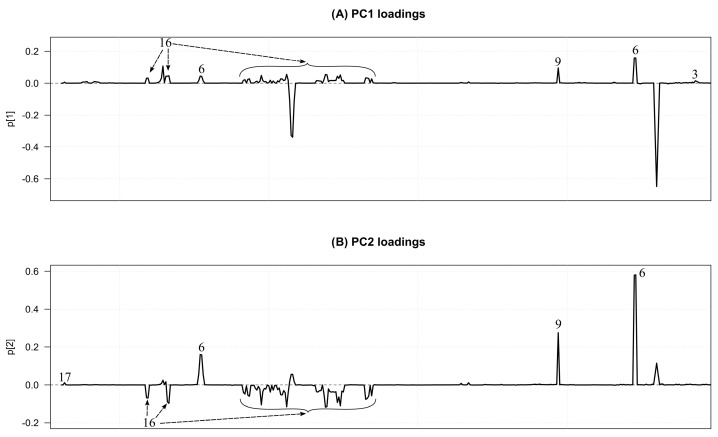
Loading plots of the PCA model of the full *S. aureus* sample set: PC1 and PC2 loadings with the x-axis reversed to mimic the chemical shift axis of a ^1^H NMR spectrum. Significant metabolites and their labels are presented in Table 1.

Metabolites with the most contribution to the principal components of the PCA model shown in Figure 5A were identified from the associated model loadings (Appendix A Figure A2) and labeled in the ^1^H spectrum in Figure 6. Valine, lactate, and acetate showed positive contributions in the loading plot, while glucose and formate showed negative contributions. There were fewer metabolites that were found to be significant in this set than in the *E. coli* set, again highlighting that not as much was changing in the metabolome overall and therefore supporting the previous idea that not as much bacterial growth was occurring due to the strong initial inhibition. 

**Figure 7:**
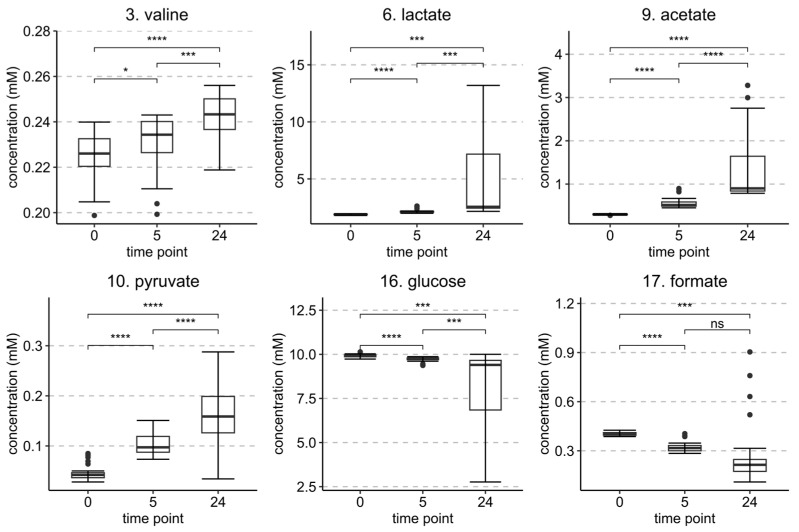
Statistically significant (*p* < 0.05) identified metabolites and their concentrations from the *S. aureus* sample set at 0 h, 5 h, and 24 h time points. Metabolites are numbered according to Table 1. *p* values of difference are from a paired *t*-test (*n* = 30). Levels of significance: **** *p* < 0.0001; *** *p* < 0.001; * *p* < 0.05; ns = non-significant.

Statistically significant changes in metabolite concentrations between the time points of 0 h, 5 h, and 24 h are shown in Figure 7 and for the same time intervals but with separated HT treatments in the Supplementary Materials in Figure S2. Glucose levels decreased in both monitored intervals. Compared to the *E. coli* set, the slower rate of decrease observed here suggests that the growth of *S. aureus* was more efficiently inhibited by the HT treatments. Some microbial growth was, however, taking place as the concentrations of lactate and acetate increased significantly over time. The separated changes in metabolite concentrations of different HT treatments in Figure S2 show that the HT-treated samples produced much less lactate and acetate than the control samples, supporting the observation of efficient inhibition. Differences between the HT treatments in the concentration changes of lactate and acetate were observed in the following order starting from the one with the highest lactate and acetate production, i.e., the least effective in inhibiting microbial growth: strictinin (**1**), castalagin (**2**), pentagalloylglucose (**4**), tellimagrandin II (**3**), and rugosin D (**6**). This order of effectiveness is the same that was observed earlier for the *E. coli* set, but there the order could be detected also directly from the PCA plots of the data rather than only from concentration changes of individual metabolites. 

An interesting observation was that the inhibition capacity of samples treated with strictinin or castalagin seemed to decline over time as the amount of lactate and acetate that was produced in those samples was close to the control samples at the later 24 h time point. However, HTs with stronger inhibitive efficiency, i.e., pentagalloylglucose (**4**), tellimagrandin II (**3**), and rugosin D (**6**), were able to maintain their inhibition efficiency until the 24 h time point. Another noteworthy observation in this *S. aureus* set is that pentagalloylglucose seems to maintain its inhibition efficiency better over time than castalagin in comparison to the results obtained for *E*. *coli*. This suggests that even though the two HTs can initially influence the bacterial culture metabolomes similarly, the type of structure of pentagalloylglucose might be more resilient for a longer inhibition.

#### 3.1.3. Metabolomic Alterations in Fecal Batch Cultures with Ampicillin-Resistant *S. aureus*

**Figure 8:**
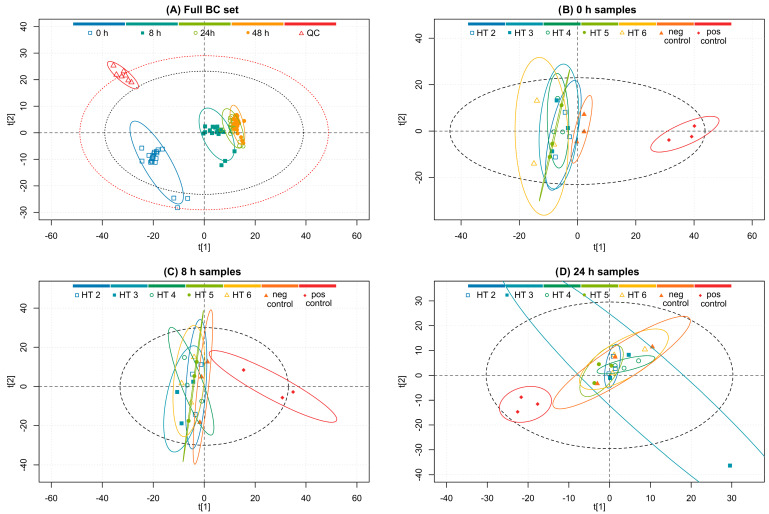
PCA model score plots of the (**A**) full fecal BC sample set (R^2^X(1) = 0.38, R^2^Y(2) = 0.13), (**B**) samples at the 0 h time point (R^2^X(1) = 0.40, R^2^Y(2) = 0.11), (**C**) samples at the 8 h time point (R^2^X(1) = 0.23, R^2^Y(2) = 0.19), and (**D**) samples at the 24 h time point (R^2^X(1) = 0.19, R^2^Y(2) = 0.18). Plot (**A**) groups according to the time point or quality control (QC), and plots (**B**–**D**) groups according to the HT treatment or control. Red and grey ellipses show Hotelling’s T^2^ 99% and 95% confidence intervals, respectively, and group ellipses show 95% confidence intervals. (**A**) 0 h samples (□, dark blue), 8 h samples (■, dark green), 24 h samples (⚪, light green), 48 h samples (⚫, orange), and quality control samples (△, red). (**B**–**D**) HT 2, castalagin (□, dark blue); HT 3, tellimagrandin II (■, turquoise); HT 4, pentagalloylglucose (⚪, dark green); HT 5, salicarinin A (⚫, light green); HT 6, rugosin D (△, yellow); negative control (▲, orange); and positive control (♦, red).

The third studied bacterial culture was more complex than the purely bacterial *E. coli* and *S. aureus* cultures because it contained the microbiota from the donors’ fecal samples as well as the added ampicillin-resistant *S. aureus* strain. Based on the results obtained from the purely bacterial sets, it was decided to replace strictinin (**1**) with salicarinin A (**5**) due to strictinin’s ineffectiveness in the bacterial set experiments. Salicarinin A is an acyclic dimeric HT that has been reported to inhibit bacterial growth effectively [13]. The processing applied to this sample set was similar to that for the previous two, and Figure 8A presents a PCA plot constructed from the buckets of the full spectral region of *δ* 0.02–10.10 ppm excluding only the region of suppressed water *δ* 4.7–5.0 ppm. The PCA plot groups are colored according to time points and QC samples. A much better separation between time points was observed here than in the *S. aureus* set. The separation is visually more similar to the separation witnessed in the *E. coli* set. The reason behind this similarity becomes more evident when the results of plated bacterial growth measurements and the bacterial sequencing are considered more in depth in the following Section 3.2 and Section 3.3.

The effects of the different HT treatments are displayed in the time-point-separated PCA plots in Figure 8B–D. In this batch culture, the longer observation time of 48 h was included to observe possible long-term changes. However, the 48 h time point was omitted from the PCA plots as there was no longer any observable change in the metabolome and the amount of variance explained by the principal components of a PCA model on the 48 h samples was low. Interestingly, the HT treatments initially show decent separation; i.e., different HTs exerted varying changes to the studied metabolome. However, this observed separation was quickly lost in the successive time points, and in the 24 h samples, all clusters of five different HT treatments were completely overlapping. 

**Figure 9:**
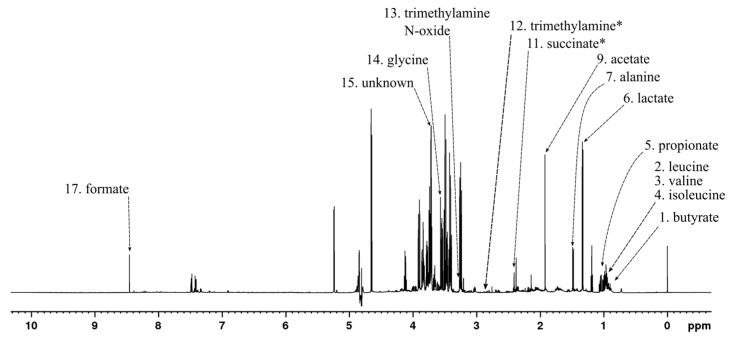
^1^H NMR spectrum of fecal BC culture sample at the 0 h time point after treatment with rugosin D with example signals of the significant metabolites labeled according to Table 1. * Marked metabolite was not visible with used scale in the 0 h sample.

**Figure A3:**
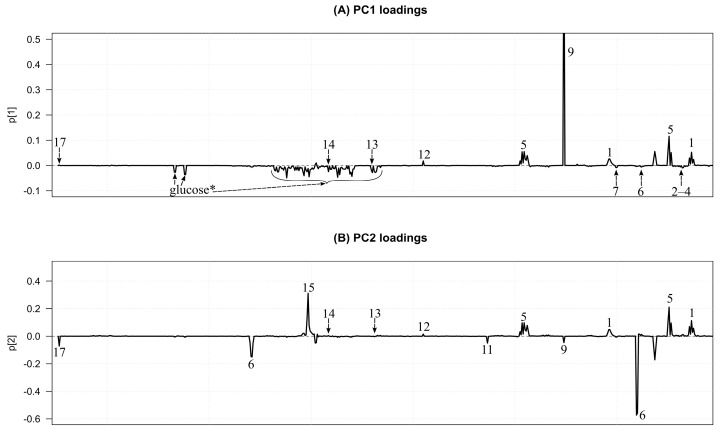
Loading plots of the PCA model of the full fecal batch culture sample set: PC1 and PC2 loadings with the x-axis reversed to mimic the chemical shift axis of a ^1^H NMR spectrum. Significant metabolites and their labels are presented in Table 1. * Glucose marked in addition to the significant metabolites due to its prevalence in the graph.

The loading plot (Appendix A Figure A3) of the PCA plot in Figure 8A reveals metabolites with significant contributions to the principal components. Altogether, 15 significant metabolites were observed, and they are labeled in the ^1^H NMR spectrum in Figure 9. Strong positive contributions to the principal components were observed for butyrate, propionate, acetate, and one unassigned metabolite, while smaller positive ones were observed for trimethylamine and glycine. Strong negative contributions were observed for lactate and formate, while smaller negative ones were observed for leucine, valine, isoleucine, alanine, succinate, and trimethylamine N-oxide.

**Figure 10:**
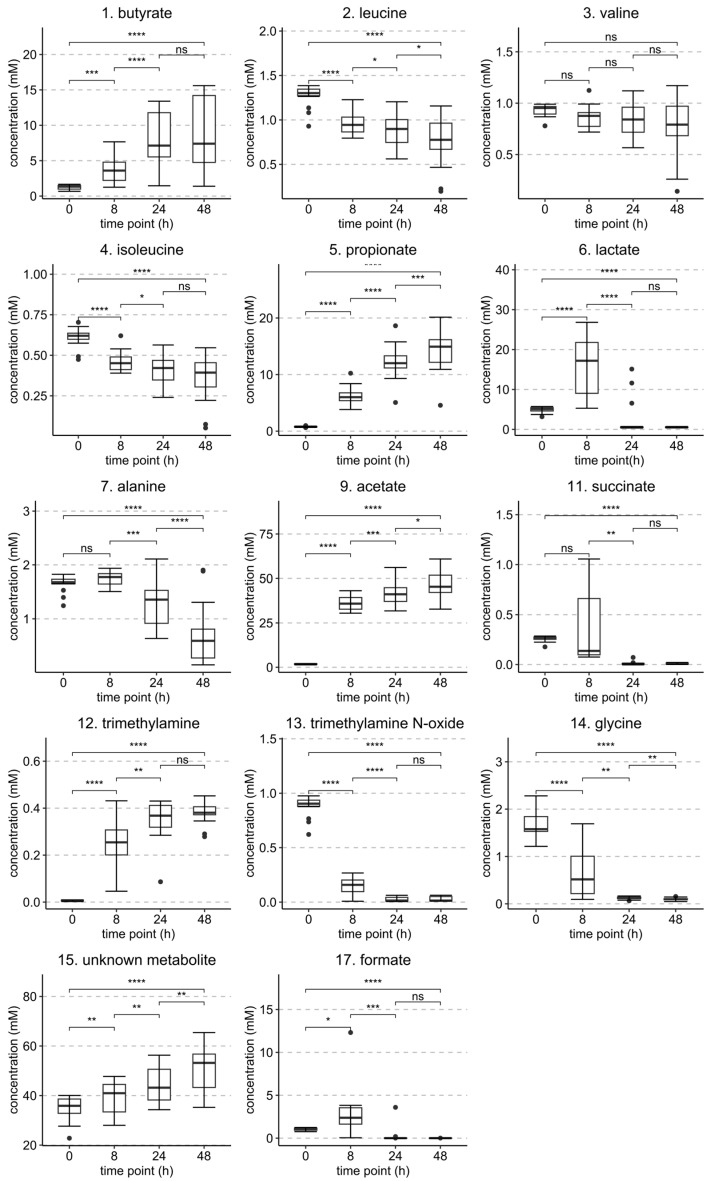
Statistically significant (*p* < 0.05) identified metabolites and their concentrations from the fecal BC sample set at 0 h, 8 h, 24 h, and 48 h time points. Metabolites are numbered according to Table 1. *p* values of difference are from a paired *t*-test (*n* = 30). Levels of significance: **** *p* < 0.0001; *** *p* < 0.001; ** *p* < 0.01; * *p* < 0.05; ns = non-significant.

Statistically significant changes in metabolite concentrations between the time points of 0 h, 8 h, 24 h, and 48 h are shown in Figure 10 and for the same time points but with separated HT treatments in the Supplementary Materials in Figures S3 and S4. Most of the changes in the metabolite concentrations between the 24 h and 48 h samples were non-significant.

Glucose concentrations were not monitored in this set because they declined in the first interval to non-detectable levels suggesting either high microbial growth or otherwise quick glucose degradation in the samples. Butyrate, propionate, acetate, trimethylamine, and the unknown metabolite increased in both 0 h to 8 h and 8 h to 24 h intervals. However, the concentrations of lactate and formate increased only in the first monitored interval, while succinate concentration did not increase significantly even in that instance. The rapidness of the metabolic changes made it challenging to establish differences between the different HT treatments as the most significant changes occurred in the first monitored interval. However, the metabolite changes in all of the HT-treated samples were significantly different compared to the positive control samples. Leucine, valine, isoleucine, alanine, trimethylamine N-oxide, and glycine concentrations decreased in most of the observed intervals.

### 3.2. Bacterial Growth Inhibition from Plate Measurements

**Figure 11:**
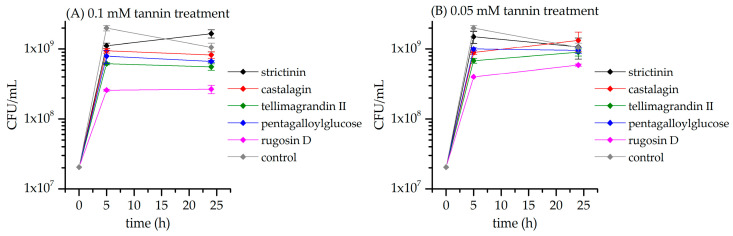
Growth of *E. coli* 5 h and 24 h after treatment with hydrolyzable tannin. Control culture was not treated with hydrolyzable tannins. Bacterial growth is presented as colony-forming units (CFUs) per mL (mean and standard error, *n* = 3).

The bacterial growth of the time point samples of *E. coli* and *S. aureus* was measured with plating experiments. The aim was to observe if the bacterial inhibition efficiency of the different HTs varied after 5 and 24 h of incubation and also if different HT concentrations changed the inhibition characteristics. Figure 11A shows the growth of *E. coli* in the presence of 0.1 mM solutions of HTs **1**–**4** and **6**. All of the studied HTs inhibited the growth of *E. coli* at the 5 h time point compared to the control culture. The displayed order of inhibition is in line with what was earlier observed from the multivariate analysis of the NMR data and the concentration changes of the individual metabolites. HTs were in the following order from the weakest to strongest inhibitor: strictinin, castalagin, pentagalloylglucose, tellimagrandin II, and rugosin D. All other HT-treated samples, except strictinin, showed either slow decline or stability in the amount of plated colony forming units (CFUs) moving from the 5 h samples to the 24 h samples. The growth of *E. coli* seemed to be even higher in the strictinin-treated samples 24 h after the treatment than in the control samples. 

The lower HT concentration used, 0.05 mM, (Figure 11B) displayed similar trends in the order of strength of *E. coli* inhibition with the least inhibition observed for strictinin and the most for rugosin D. However, with the lower HT concentration, the trend in CFUs moving from the 5 h samples to the 24 h samples was an increasing one with the samples treated with castalagin, tellimagrandin II, and rugosin D. This trend suggested that the 0.05 HT solutions could not inhibit the growth of *E. coli* as efficiently as 0.1 mM solutions when monitored for longer. Interestingly, the lower concentration of strictinin exhibited more inhibition than the higher concentration in the 24 h samples.

**Figure 12:**
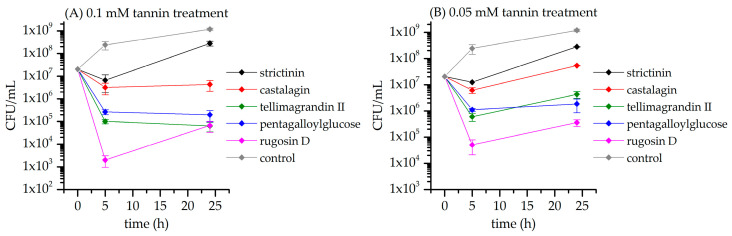
Growth of *S. aureus* 5 h and 24 h after treatment with hydrolyzable tannin. Control culture was not treated with hydrolyzable tannins. Bacterial growth is presented as colony-forming units (CFUs) per mL (mean and standard error, *n* = 3).

Figure 12 shows the growth of *S. aureus* in the presence of HTs **1**–**4** and **6** with two different concentrations. Compared to the control sample, the CFU values of the HT-treated samples are several orders of magnitude smaller with both of the studied HT concentrations at the 5 h time point. The same order of inhibition efficacy of HTs was observed as earlier for the NMR metabolomic dataset. The results also in part revealed why the NMR metabolomics provided little separation between the clusters of time point samples of *S. aureus*. The observed lack of separation was in part due to all of the studied HTs inhibiting the *S. aureus* so effectively that their plated CFU values decreased from the 0 h samples. This suggests that when bacterial growth is heavily inhibited, the bacterial metabolome does not change noticeably over time. The plating results of the time point samples of *S. aureus* were observed to be in line with previously reported antimicrobial results [13]. 

Similar plating experiments were also performed for the fecal BC set samples, but during the culture growth, some challenges were encountered. Ampicillin-resistant *S. aureus* was added to the fecal BC set samples to see how the HTs can inhibit *S. aureus* in this more biomimetic environment with the fecal samples included in the mixture. The plating experiments were performed with ampicillin-dosed plates where the ampicillin was intended to kill all other bacteria that might have been present due to the fecal samples. This, however, was not successful, as two of the three fecal sample donors had previously had multiple ampicillin treatments. Thus, some ampicillin-resistant bacteria from the *Enterobacteriaceae* family (see Section 3.3) might have unfortunately also been present in the samples, which grew to such an extent that any colony counting was rendered impossible.

### 3.3. Bacterial DNA Sequencing

DNA sequencing of the fecal BC set samples revealed new insights into how the relative proportions of different bacteria changed in the samples. Changes were observed according to the time points of sample collection, the different HT treatments, and between the different replicates, i.e., the three fecal sample donors. 

**Figure A4:**
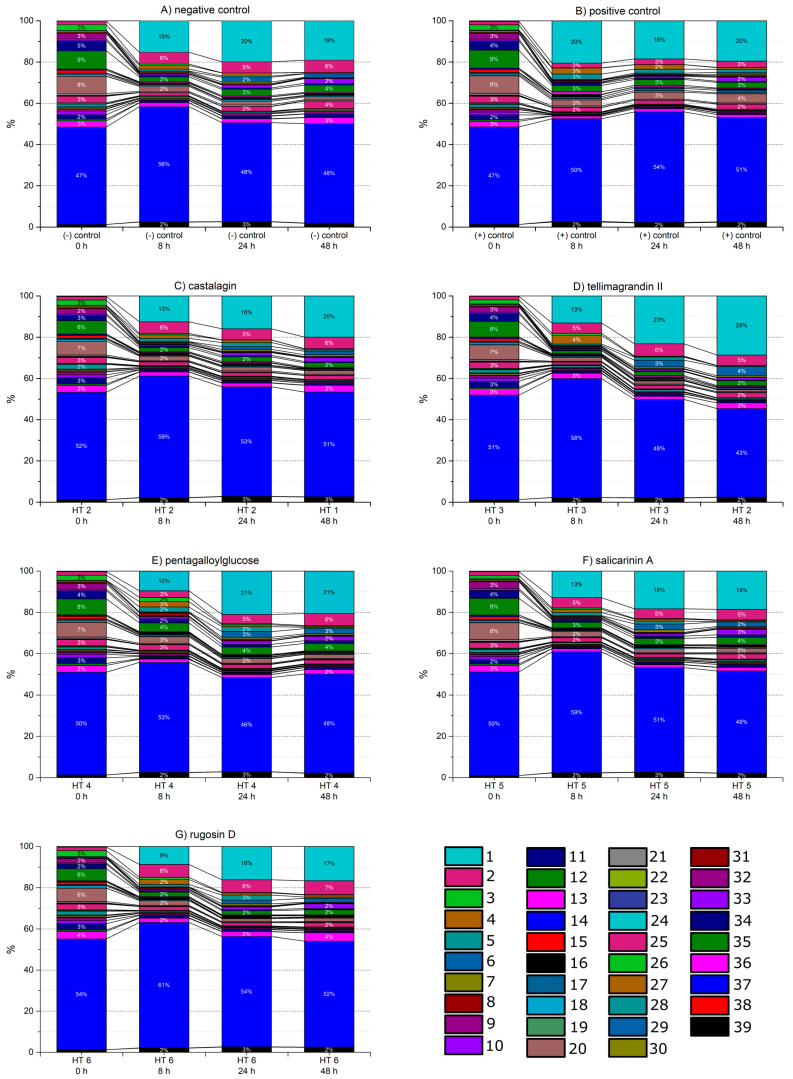
Bacterial DNA sequencing results of the fecal batch culture sample set treated with different hydrolyzable tannins (HTs). (**A**) Negative control, (**B**) positive control, (**C**) HT 2 = castalagin, (**D**) HT 3 = tellimagrandin II, (**E**) HT 4 = pentagalloylglucose, (**F**) HT 5 = salicarinin A, and (**G**) HT 6 = rugosin D treated samples. Labels annotated for sequences with more than 1% relative concentration. Legend titles (sequenced bacterial order, family, genus): (1) Enterobacteriales, *Enterobacteriaceae*, –; (2) Burkholderiales, *Alcaligenaceae*, *Sutterella*; (3) RF32, –, –; (4) Erysipelotrichales, *Erysipelotrichaceae*, *Catenibacterium*; (5) Erysipelotrichales, *Erysipelotrichaceae*, –; (6) Clostridiales, *Veillonellaceae*, *Veillonella*; (7) Clostridiales, *Veillonellaceae*, *Dialister*; (8) Clostridiales, *Veillonellaceae*, –; (9) Clostridiales, *Ruminococcaceae*, *Ruminococcus*; (10) Clostridiales, *Ruminococcaceae*, *Oscillospira*; (11) Clostridiales, *Ruminococcaceae*, *Faecalibacterium*; (12) Clostridiales, *Ruminococcaceae*, –; (13) Clostridiales, *Lachnospiraceae*, *Ruminococcus*; (14) Clostridiales, *Lachnospiraceae*, *Roseburia*; (15) Clostridiales, *Lachnospiraceae*, *Lachnospira*; (16) Clostridiales, *Lachnospiraceae*, *Dorea*; (17) Clostridiales, *Lachnospiraceae*, *Coprococcus*; (18) Clostridiales, *Lachnospiraceae*, *Blautia*; (19) Clostridiales, *Lachnospiraceae*, *Anaerostipes*; (20) Clostridiales, *Lachnospiraceae*, –; (21) Clostridiales, *Lachnospiraceae*, Other; (22) Clostridiales, *Clostridiaceae*, *Clostridium*; (23) Clostridiales, *Clostridiaceae*, –; (24) Clostridiales, *Christensenellaceae*, –; (25) Clostridiales, –, –; (26) Clostridiales, Other, Other; (27) Lactobacillales, *Streptococcaceae*, *Streptococcus*; (28) Bacillales, *Staphylococcaceae*, *Staphylococcus*; (29) YS2, –, –; (30) Bacteroidales, *Odoribacteraceae*; *Odoribacter*; (31) Bacteroidales, *Odoribacteraceae*; *Butyricimonas*; (32) Bacteroidales, *Barnesiellaceae*; –; (33) Bacteroidales, S24-7, –; (34) Bacteroidales, *Rikenellaceae*, –; (35) Bacteroidales, *Prevotellaceae*, *Prevotella*; (36) Bacteroidales, *Porphyromonadaceae*, *Parabacteroides*; (37) Bacteroidales, *Bacteroidaceae*, *Bacteroides*; (38) Bifidobacteriales, Bifidobacteriaceae, Bifidobacterium; (39) Unassigned.

Figure A4 shows the time-evolved bacterial metabolome with relative concentration percentages from the samples treated with five HTs (**2**–**6**), the negative controls, and the positive controls. The 0 h time point samples refer to the starting microbiota, from the initial donor samples. The HT treatments did not have a significant impact on the microbial community relative to the negative control. However, when looking at all samples, including positive and negative controls, two notable bacterial concentration changes were observed. Firstly, *Staphylococcus* was initially detectable in the 0 h samples, as expected due to *S. aureus* being added to the vessels. However, at the other fermentation time points, *Staphylococcus* was present only in low proportions; therefore, regardless of the HTs, the *Staphylococcus* seemed unable to survive well within the batch environment. Secondly, bacteria levels from the family *Enterobacteriaceae* increased from practically non-detectable in the 0 h samples to between 12% and 30% at later time points. These two observations could reinforce what was earlier noticed in the plating experiments where *E. coli*, part of the *Enterobacteriaceae*, was observed to grow intensely instead of the added *S. aureus*. Upon closer inspection, the increase in the *Enterobacteriaceae* bacteria was only present in the samples with the fecal material from the two donors who were known to have undergone multiple ampicillin treatments before (data not shown). Obtained results were hard to interpret, as the effect of HT treatment was minor in comparison to the effect of the discrepancy between the donors’ fecal sample microbial contents.

## 4. Conclusions

The studied HTs modified the metabolome of *E. coli* and *S. aureus* cultures with observable differences between sample collections at different time points and between different HT treatments. The structural features of HTs were found to influence the degree of metabolomic modification they were able to exert. The impact of these structural features was further confirmed when the concentration changes of individual metabolites were examined. Between the different HT treatments, notable differences were observed in changes in acetate, formate, lactate, and succinate concentrations. The concentrations of these overflow metabolites increased with time, suggesting that some bacterial growth was still taking place. Effective HT structural features included having a cyclic polyol and multiple free galloyl groups, both of which increase the hydrophobicity of the structure and thus increase the probability that these structures can interact with and permeate bacterial lipid membranes. Interestingly, it was also discovered that some structural features that decrease hydrophobicity such as the presence of HHDP groups instead of two free galloyl groups in a structure increased the efficiency of bacterial growth inhibition. The most effective of the studied HTs was rugosin D, a dimeric tannin with cyclic polyols and multiple free galloyl groups and HHDP groups in its structure. Rugosin D can be found abundantly in some plants of the *Rosaceae* family, such as *Rosa rugosa* and *Filipendula ulmaria* [60,61]. The same plants also produce the second most effective HT in this study, tellimagrandin II, in large quantities.

Plated inhibition results supported the order of efficacy of HTs observed from the NMR metabolomic data, verifying that the observed metabolomic changes corroborated bacterial inhibition. The inhibition results were in line with previously reported ones and also in line with the literature; studied HTs were more effective in inhibiting *S. aureus* than *E. coli*. 

## Figures and Tables

**Table 1:** Identified significant metabolites from the *E. coli*, *S. aureus*, and fecal BC sample sets, selected resonance ^1^H chemical shifts (ppm), multiplicities, and the number of protons contributing to these resonances.

# and Presence in Each Set ^a^			Metabolite	Chemical Shift ^b^ (ppm)
*E. coli*	*S. aureus*	Fecal BC		
nd	nd	1	butyrate	0.88 (t, 3H), 1.55 (m, 2H)
2	nd	2	leucine	0.95 (t, 6H),
3	3	3	valine	0.98 (d, 3H), 1.03 (d, 3H)
nd	nd	4	isoleucine	1.00 (d, 3H)
nd	nd	5	propionate	1.04 (t, 3H), 2.17 (q, 2H)
6	6	6	lactate	1.32 (d, 3H), 4.10 (q, 1H)
nd	nd	7	alanine	1.47 (d, 3H), 3.77 (q, 1H)
8	nd	nd	lysine	1.73 (p, 2H), 3.03 (t, 2H)
9	9	9	acetate	1.91 (s, 3H)
10	10	nd	pyruvate	2.36 (s, 3H)
11	nd	11	succinate	2.40 (s, 4H)
nd	nd	12	trimethylamine	2.87 (s, 9H)
nd	nd	13	trimethylamine N-oxide	3.27 (s, 9H)
nd	nd	14	glycine	3.55 (s, 2H)
nd	nd	15	unknown ^c^	3.71 (m)
16	16	ns	glucose	4.65 (d, 1H), 5.23 (d, 1H)
17	17	17	formate	8.44 (s, 1H)

^a^ nd = not detected, ns = non-significant. ^b^ Well-resolved metabolite signals used for metabolite concentration calculations. Multiplicity: singlet (s), doublet (d), triplet (t), quartet (q), pentet (p), undefined multiplet (m). ^c^ Metabolite not identified.

## Data Availability

The data presented in this study are available on request from the corresponding author due to privacy or ethical restrictions.

## Supplementary Materials

The following supporting information can be downloaded at: https://www.mdpi.com/article/10.3390/metabo13030320/s1, Figure S1: Statistically significant (*p* < 0.05) identified metabolites and their concentrations from the *E. coli* sample set at 0 h, 5 h, and 24 h time points with hydrolyzable tannin (HT) treatments and controls separated; Figure S2: Statistically significant (*p* < 0.05) identified metabolites and their concentrations from the *S. aureus* sample set at 0 h, 5 h, and 24 h time points with hydrolyzable tannin (HT) treatments and controls separated; Figure S3: Statistically significant (*p* < 0.05) identified metabolites (1–7, 9, and 11) and their concentrations from the fecal BC sample set at 0 h, 5 h, 24 h, and 48 h time points with hydrolyzable tannin (HT) treatments and controls separated; Figure S4: Statistically significant (*p* < 0.05) identified metabolites (12–15 and 17) and their concentrations from the fecal BC sample set at 0 h, 5 h, 24 h, and 48 h time points with hydrolyzable tannin (HT) treatments and controls separated; S1: Hydrolyzable tannins utilized in the study with information on plant origin, purity measured by UPLC-DAD at 280 nm, ESI-MS identification, and ^1^H NMR assignation with labeled chemical structure.
