# Supplementary material for: NMR Metabolomics and DNA Sequencing of Escherichia coli and Staphylococcus aureus Cultures Treated with Hydrolyzable Tannins
Source: Metabolites. 2023 Feb 21;13(3):320. doi: 10.3390/metabo13030320 (PMC10051465; doi:10.3390/metabo13030320)
Supplement: Supplementary file 1 [file metabolites-13-00320-s001.zip › metabolites-2221306-supplementary.pdf]

metabolites

MDPI

Supplementary Materials

NMR metabolomics and DNA sequencing of *Escherichia coli* and *Staphylococcus aureus* cultures treated with hydrolysable tannins

Valtteri Virtanen [1,*], Elina Puljula [1], Gemma Walton [2], Martin J. Woodward [2,3] and Maarit Karonen [1]

[1] Natural Chemistry Research Group, Department of Chemistry, University of Turku, FI-20014 Turku, Finland;
[2] Department of Food and Nutritional Studies, The University of Reading, Reading RG6 6AH, UK
[3] Folium Science, Science Creates, Midland Road, Old Market, Bristol, BS2 0JZ
* Correspondence: vtjvir@utu.fi; Tel.: +358-29-450-3205

Table of Contents

Figure S1. Statistically significant (P < .05) identified metabolites and their concentrations from the E. coli sample set at 0 h, 5 h, and 24 h time points with hydrolysable tannin (HT) treatments and controls separated................................ 2
Figure S2. Statistically significant (*P* < .05) identified metabolites and their concentrations from the *S. aureus* sample set at 0 h, 5 h, and 24 h time points with hydrolysable tannin (HT) treatments and controls separated.......................... 3
Figure S3. Statistically significant (*P* < .05) identified metabolites (1–7, 9 and 11) and their concentrations from the fecal BC sample set at 0 h, 5 h, 24 h, and 48 h time points with hydrolysable tannin (HT) treatments and controls separated.. 4
Figure S4. Statistically significant (*P* <.05) identified metabolites (12–15 and 17) and their concentrations from the fecal BC sample set at 0 h, 5 h, 24 h, and 48 h time points with hydrolysable tannin (HT) treatments and controls separated.. 5
S1. Hydrolysable tannins utilized in the study with information on plant origin, purity measured by UPLC-DAD at 280 nm, ESI-MS identification and ^{1}H NMR assignation with labeled chemical structure.. 5

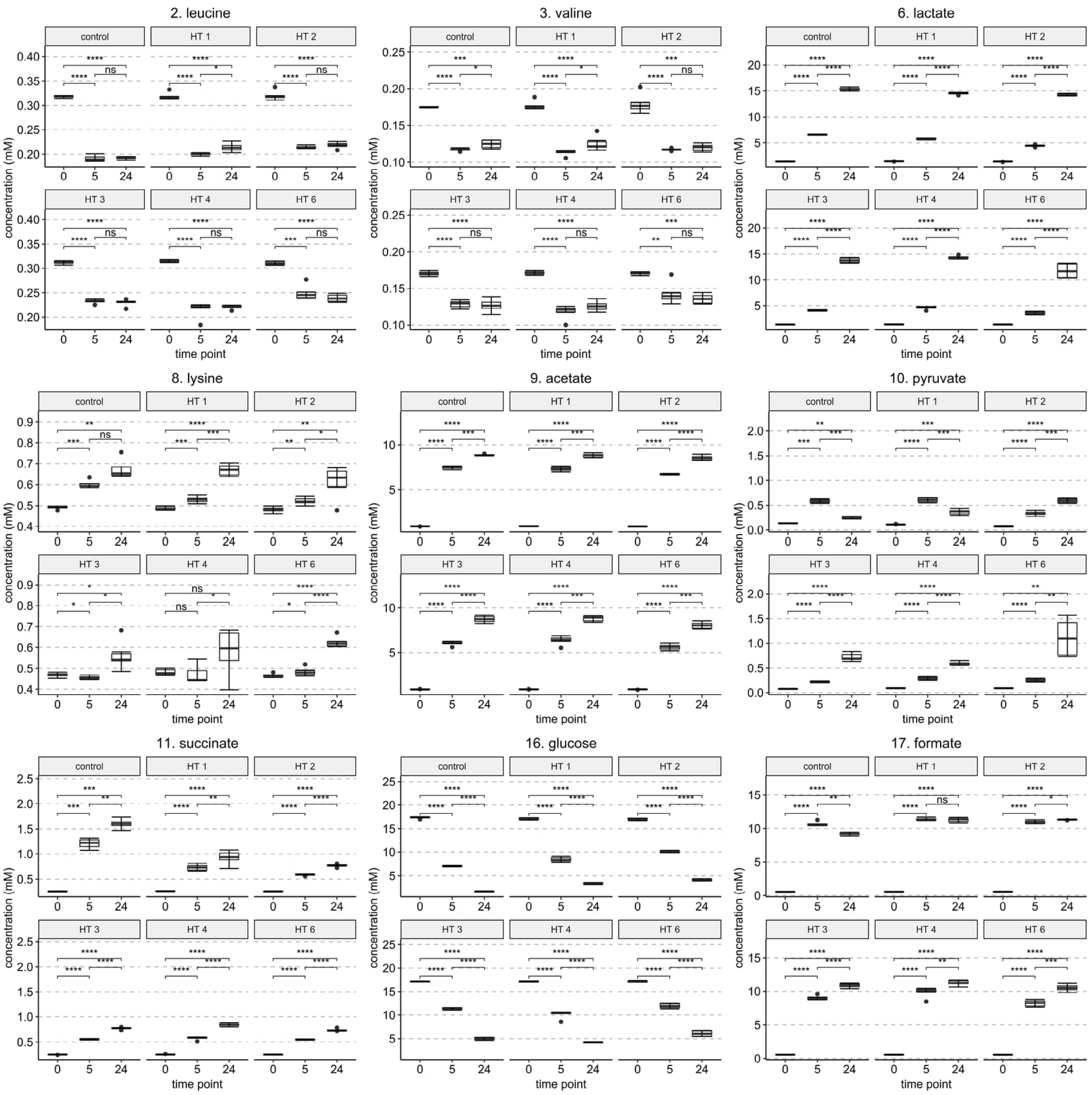

Figure S1. Statistically significant (P < .05) identified metabolites and their concentrations from the E. coli sample set at 0 h, 5 h, and 24 h time points with hydrolysable tannin (HT) treatments and controls separated. HT 1, strictinin; HT 2, castalagin; HT 3, tellimagrandin II; HT 4, pentagalloylglucose; HT 6, rugosin D. Metabolites are numbered according to Table 1 in the article. The y-axis of the subplots of each metabolite is scaled according to the largest observed concentration of that metabolite. Levels of significance: **** *P*<.0001; *** *P*<.001; ** *P*<.01; * *P*<.05; ns=non-significant. *P* values of difference are from a paired t-test (n=6).

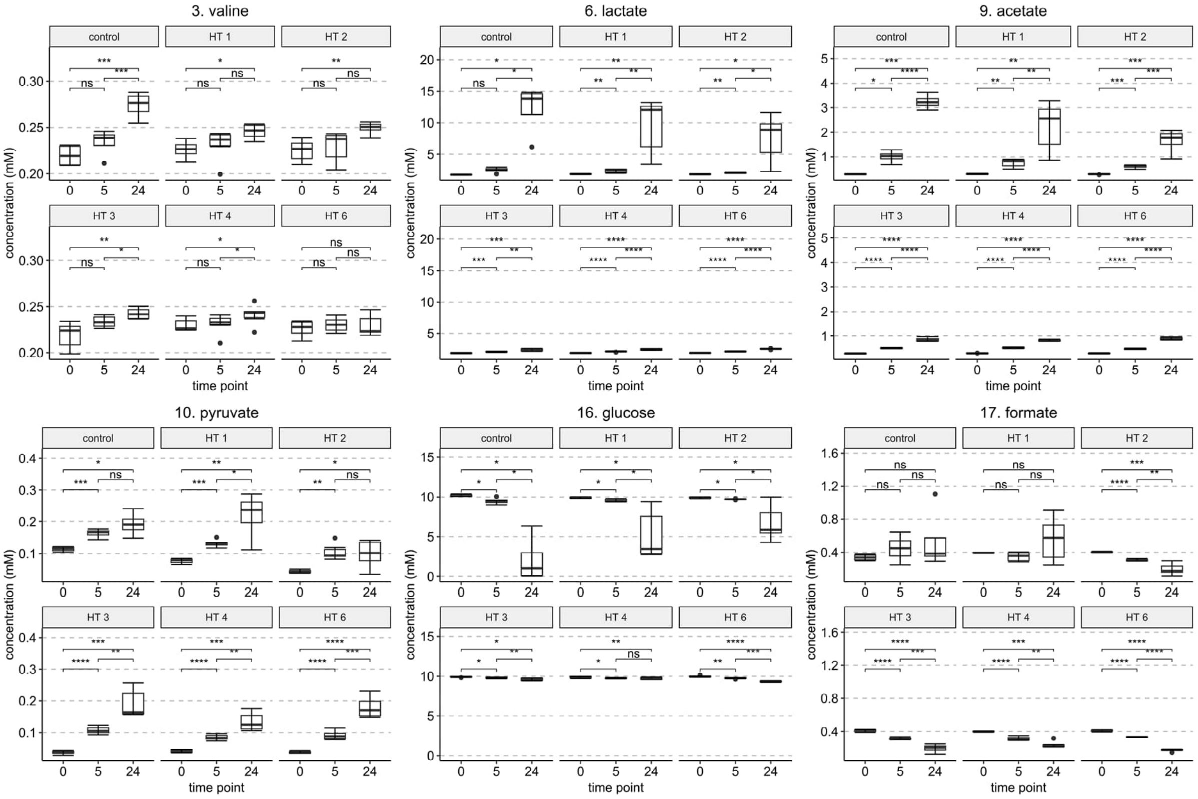

Figure S2. Statistically significant ($P < .05$) identified metabolites and their concentrations from the *S. aureus* sample set at 0 h, 5 h, and 24 h time points with hydrolysable tannin (HT) treatments and controls separated. HT 1, strictinin; HT 2, castalagin; HT 3, tellimagrandin II; HT 4, pentagalloylglucose; HT 6, rugosin D. Metabolites are numbered according to Table 1 in the article. The y-axis of the subplots of each metabolite is scaled according to the largest observed concentration of that metabolite. Levels of significance: **** *P*<.0001; *** *P*<.001; ** *P*<.01; * *P*<.05; ns=non-significant. *P* values of difference are from a paired t-test (n=6).

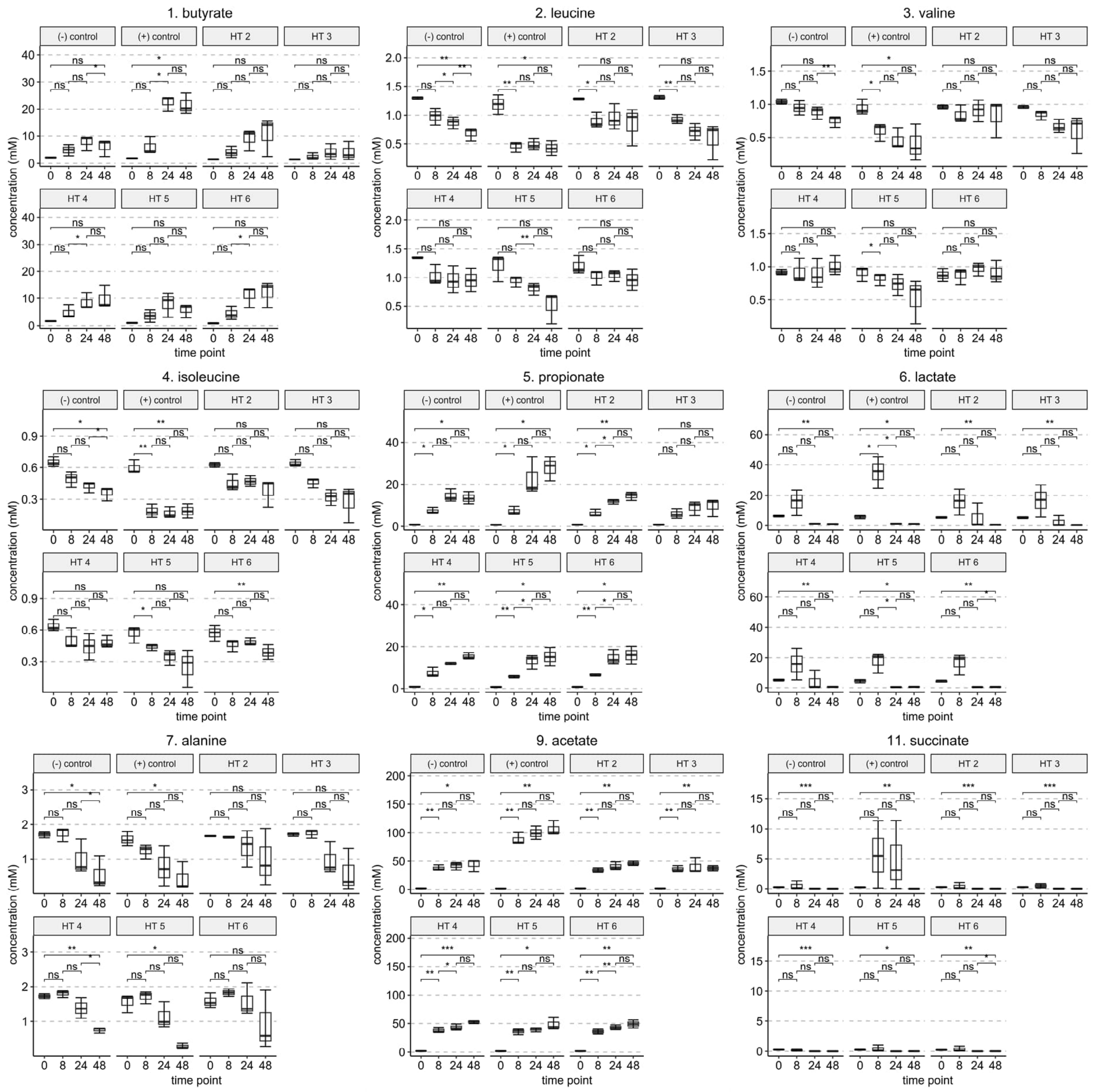

Figure S3. Statistically significant (*P* < .05) identified metabolites (1–7, 9 and 11) and their concentrations from the fecal BC sample set at 0 h, 5 h, 24 h, and 48 h time points with hydrolysable tannin (HT) treatments and controls separated. HT 2, castalagin; HT 3, tellimagrandin II; HT 4, pentagalloylglucose; HT 5, salicarinin A; HT 6, rugosin D. Metabolites are numbered according to Table 1 in the article. The y-axis of the subplots of each metabolite is scaled according to the largest observed concentration of that metabolite. Levels of significance: **** *P*<.0001; *** *P*<.001; ** *P*<.01; * *P*<.05; ns=non-significant. *P* values of difference are from a paired t-test (n=3).

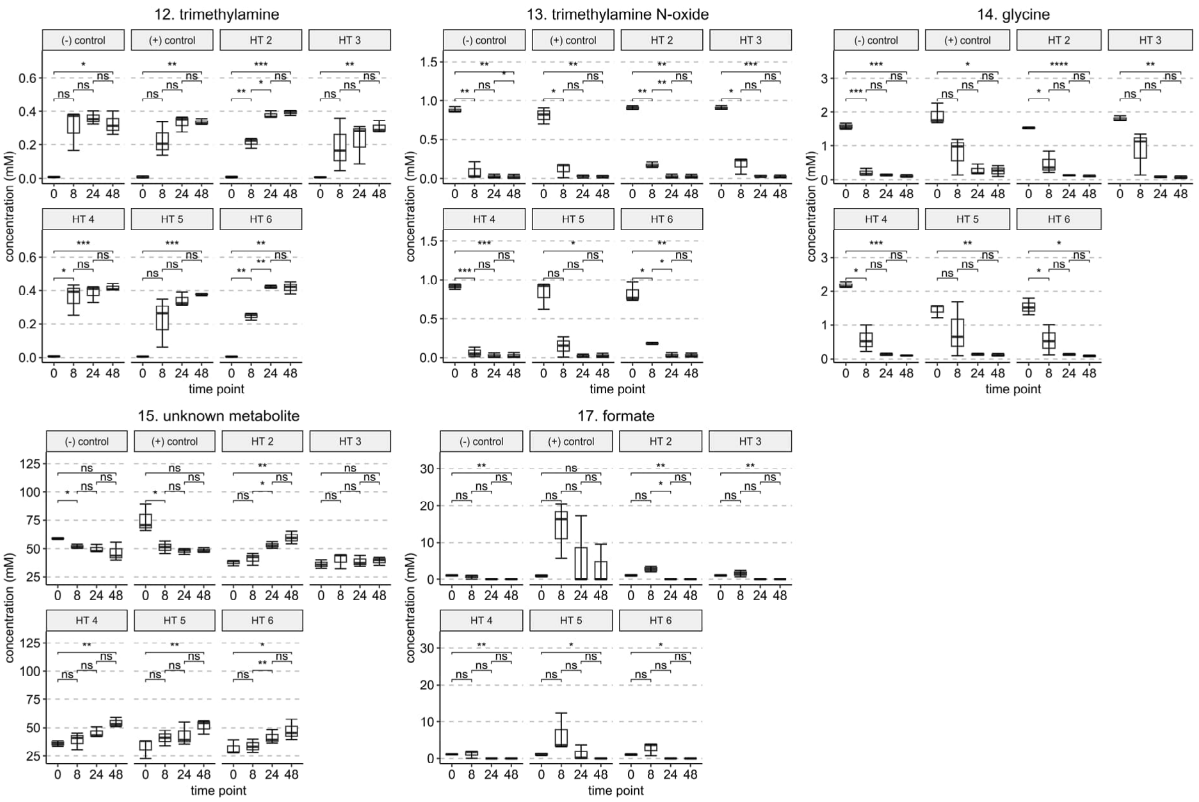

Figure S4. Statistically significant (P <.05) identified metabolites (12–15 and 17) and their concentrations from the fecal BC sample set at 0 h, 5 h, 24 h, and 48 h time points with hydrolysable tannin (HT) treatments and controls separated. HT 2, castalagin; HT 3, tellimagrandin II; HT 4, pentagalloylglucose; HT 5, salicarinin A; HT 6, rugosin D. Metabolites are numbered according to Table 1 in the article. The y-axis of the subplots of each metabolite is scaled according to the largest observed concentration of that metabolite. Levels of significance: **** P<.0001; *** P<.001; ** P<.01; * P<.05; ns=non-significant. P values of difference are from a paired t-test (n=3).

S1. Hydrolysable tannins utilized in the study with information on plant origin, purity measured by UPLC-DAD at 280 nm, ESI-MS identification and ^{1}H NMR assignation with labeled chemical structure.

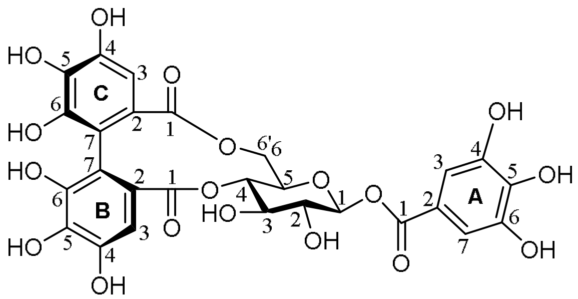

Strictinin (1) was isolated from *Hippophae rhamnoides* leaves; purity measured by UPLC-DAD at 280 nm 98.6 %; ESI-MS identification: m/z at 633.07290 ([M–H]$^-$, error –0.7 ppm), 300.99884 ([ellagic acid-H]$^-$, error –0.1 ppm); ^{1}H NMR (500 MHz, acetone-d_6, 298 K): δ 3.70 (t, 1H, J=8.5 Hz, H_{glc}-2), δ 3.78 (d, 1H, J=13.0 Hz, H_{glc}-6'), δ 3.83 (d, 1H, J=9.3 Hz, H_{glc}-3), δ 4.11 (dd, 1H, J=5.7, 9.9 Hz, H_{glc}-5), δ 4.90 (t, 1H, J=9.8 Hz, H_{glc}-4), δ 5.21 (dd, 1H, J=6.4, 13.3 Hz, H_{glc}-6), δ 5.74 (d, 1H, J=8.1 Hz, H_{glc}-1), δ 6.60 (s, 1H, H_C-3), δ 6.71 (s, 1H, H_B-3), δ 7.20 (s, 2H, H_A-3,7).

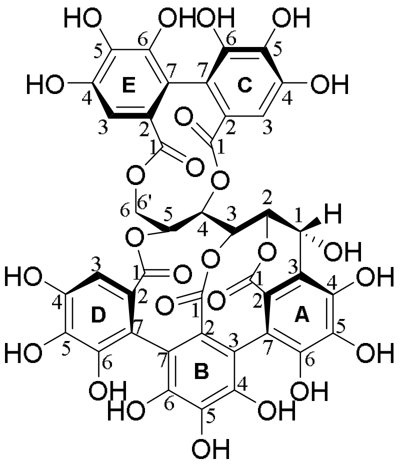

Castalagin (2) was isolated from *Quercus robur* acorns; purity measured by UPLC-DAD at 280 nm 99.6 %; ESI-MS identification: *m/z* at 933.06196 ($[M–H]^-$, error –2.1 ppm), 631.05890 ($[M–hexahydroxydiphenoyl–H]^-$, error 1.9 ppm), 300.99898 ($[ellagic\ acid–H]^-$, error –0.03 ppm); ^{1}H NMR (600 MHz, acetone-d_6, 298 K): δ 4.01 (d, 1H, J=12.5 Hz, H_{glc}-6'), δ 5.03 (m, 1H, H_{glc}-3), δ 5.04 (m, 1H, H_{glc}-4), δ 5.10 (dd, 1H, J=2.6, 13.0 Hz, H_{glc}-6), δ 5.24 (t, 1H, J=7.3 Hz, H_{glc}-4), δ 5.61 (ddd, 1H, J=1.0, 2.5, 7.7 Hz, H_{glc}-5), δ 5.71 (d, 1H, J=4.4 Hz, H_{glc}-1), δ 6.63 (s, 1H, H_E-3), δ 6.77 (s, 2H, H_C-3 & H_D-3).

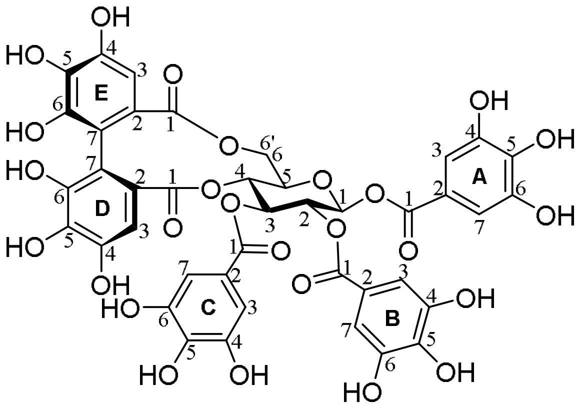

Tellimagrandin II (3) was isolated from *Filipendula ulmaria* flowers; purity measured by UPLC-DAD at 280 nm 90.2 %; ESI-MS identification: *m/z* at 937.09506 ($[M–H]^-$, error –0.2 ppm), 767.07276 ($[M–galloyl–H]^-$, error –1.3 ppm), 465.06793 ($[M–galloyl–hexahydroxydiphenoyl–H]^-$, error 1.0 ppm), 300.99885 ($[ellagic\ acid–H]^-$, error –0.5 ppm); ^{1}H NMR (600 MHz, acetone-d_6, 298 K): δ 3.89 (d, 1H, J=13.3 Hz, H_{glc}-6'), δ 4.55 (dd, 1H, J=6.4, 9.9 Hz, H_{glc}-5), δ 5.22 (t, 1H, J=10.0 Hz, H_{glc}-4), δ 5.37 (dd, 1H, J=6.6, 13.4 Hz, H_{glc}-6), δ 5.60 (dd, 1H, J=8.5, 9.4 Hz, H_{glc}-2), δ 5.85 (t, 1H, J=9.8 Hz, H_{glc}-3), δ 6.21 (d, 1H, J=8.3 Hz, H_{glc}-1), δ 6.47 (s, 1H, H_D-3), δ 6.66 (s, 1H, H_E-3), δ 6.98 (s, 2H, H_C-3,7), δ 7.01 (s, 2H, H_B-3,7), δ 7.12 (s, 2H, H_A-3,7).

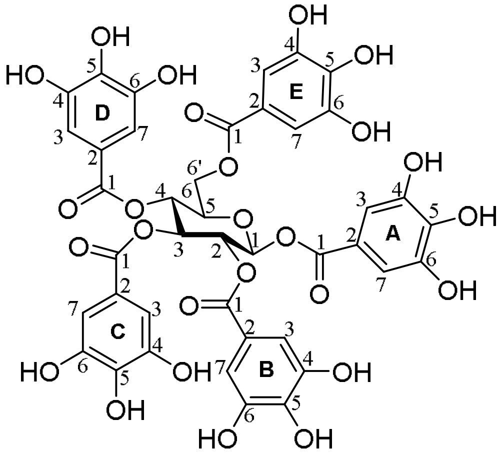

1,2,3,4,6-penta-*O*-galloyl-*β*-D-glucose (4) was purified from tannic acid (J.T. Baker, Denventer, Holland); purity measured by UPLC-DAD at 280 nm 98.6 %; ESI-MS identification: *m/z* at 939.11042 ($[M-H]^-$, error –0.5 ppm), 769.08889 ($[M-galloyl-H]^-$, error –0.6), 617.07854 ($[M-2galloyls-H]^-$, error 0.2 ppm), 169.01516 ($[gallic\ acid-H]^-$, error 5.4 ppm), 125.02547 ($[gallic\ acid-COOH-H]^-$, 8.4 ppm); ^{1}H NMR (600 MHz, acetone-d_6, 298 K): δ 4.41 (dd, 1H, J=4.5, 12.5 Hz, H_{glc}-6'), δ 4.54 (m, 1H), H_{glc}-6, δ 4.56 (ddd, 1H, J=1.6, 4.4, 9.9 Hz, H_{glc}-5), δ 5.61 (dd, 1H, J=8.3, 9.9 Hz, H_{glc}-2), δ 5.66 (t, 1H, J=9.7 Hz, H_{glc}-4), δ 6.01 (t, 1H, J=9.8 Hz, H_{glc}-3), δ 6.34 (d, 1H, J=8.3 Hz, H_{glc}-1), δ 6.97 (s, 2H, H_C-3,7), δ 7.01 (s, 2H, H_B-3,7), δ 7.06 (s, 2H, H_D-3,7), δ 7.11 (s, 2H, H_A-3,7), δ 7.18 (s, 2H, H_E-3,7).

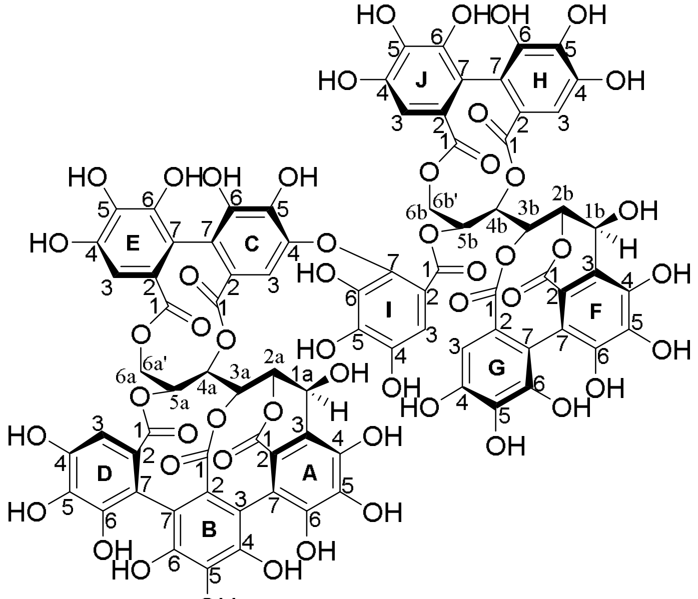

Salicarinin A (5) was isolated from *Lythrum salicaria* leaves; purity measured by UPLC-DAD at 280 nm 96.0 %; ESI-MS identification: *m/z* at 1867.13039 ($[M-H]^-$, error -2.6 ppm), 933.06290 ($[M-2H]^{2-}$, error –1.1 ppm), 924.05736 ($[M-H_2O-2H]^{2-}$, error –1.4 ppm), 915.50205 ($[M-2H_2O-2H]^{2-}$, error –1.4 ppm), 300.99885 ($[ellagic\ acid-H]^-$, error –0.4 ppm); ^{1}H NMR (600 MHz, acetone-d_6, 298 K): δ 3.89 (d, 1H, J=12.9 Hz, H_{glc}-6b'), δ 3.95 (d, 1H, J=12.5 Hz, H_{glc}-6a'), δ 4.64 (dd, 1H, J=1.1, 6.7 Hz, H_{glc}-3a), δ 4.83 (m, 2H, H_{glc}-1b & H_{glc}-2b), δ 4.85 (dd, 1H, J=3.5, 13.4 Hz, H_{glc}-6b), δ 4.93 (t, 1H, J=2.0 Hz, H_{glc}-3b), δ 4.93 (d, 1H, J=2.0 Hz, H_{glc}-1a), δ 5.08 (dd, 1H, J=2.4, 13.1 Hz, H_{glc}-6a), δ 5.14 (br s, 1H, H_{glc}-2a), δ 5.18 (t, 1H, J=6.8 Hz, H_{glc}-4a), δ 5.40 (dd, 1H, J=2.8, 8.6 Hz, H_{glc}-5b), δ 5.55 (dd, 1H, J=2.5, 8.6 Hz, H_{glc}-4b), δ 5.63 (br d, 1H, J=6.8 Hz, H_{glc}-5a), δ 6.54 (s, 1H, H_G-3), δ 6.62 (s, 2H, H_E-3 & H_J-3), δ 6.78 (s, 1H, H_D-3), δ 6.83 (s, 1H, H_C-3), δ 6.86 (s, 1H, H_H-3), δ 7.07 (s, 1H, H_I-3).

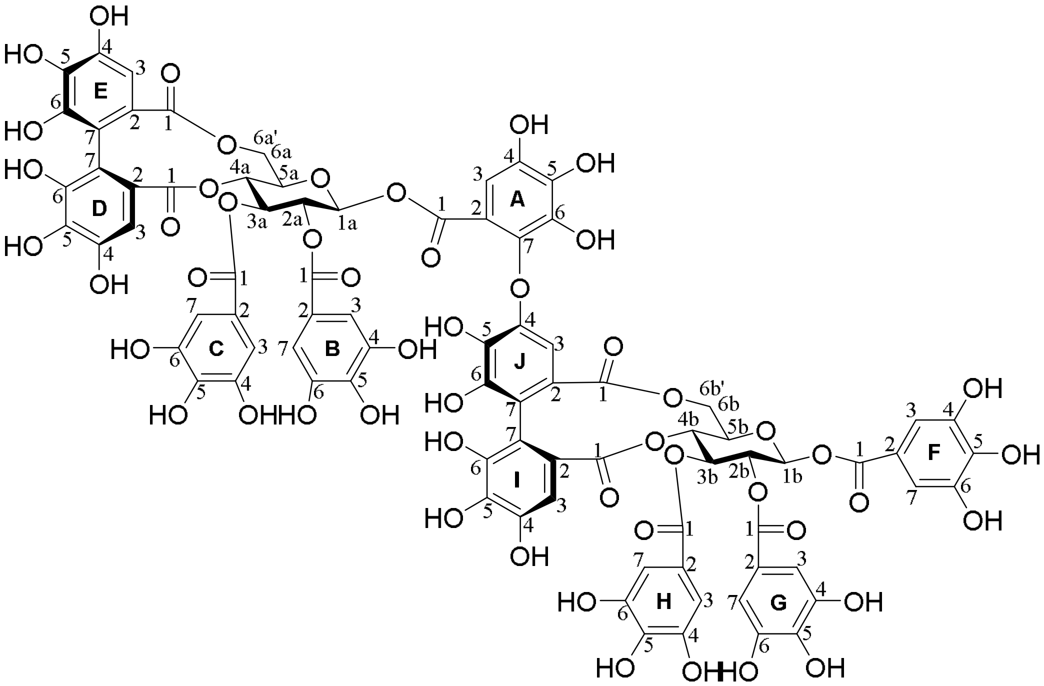

Rugosin D (6) was isolated from *Filipendula ulmaria* flowers; purity measured by UPLC-DAD at 280 nm 90.7 %; ESI-MS identification: *m*/*z* at 1873.17806 ($[M-H]^-$, error −2.1 ppm), 936.08715 ($[M-2H]^{2-}$, error −0.3 ppm), 300.99901 ([ellagic acid−H]$^-$, error 0.1 ppm); ^{1}H NMR (600 MHz, acetone-d_6, 298 K): δ 3.79 (d, 1H, J=13.4 Hz, H_{glc}-6b'), δ 3.82 (d, 1H, J=13.5 Hz, H_{glc}-6a'), δ 4.48 (dd, 1H, J=7.2, 9.9 Hz, H_{glc}-5a), δ 4.52 (dd, 1H, J=6.3, 10.0 Hz, H_{glc}-5b), δ 5.166 (t, 1H, J=10.0 Hz, H_{glc}-4b), δ 5.167 (t, 1H, J=10.0 Hz, H_{glc}-4a), δ 5.31 (dd, 1H, J=6.5, 13.4 Hz, H_{glc}-6b), δ 5.32 (dd, 1H, J=6.6, 13.4 Hz, H_{glc}-6a), δ 5.54 (dd, 1H, J=8.3, 9.5 Hz, H_{glc}-2a), δ 5.61 (dd, 1H, J=8.4, 9.5 Hz, H_{glc}-2b), δ 5.79 (t, 1H, J=9.8 Hz, H_{glc}-3a), δ 5.84 (t, 1H, J=9.8 Hz, H_{glc}-3b), δ 6.13 (d, 1H, J=8.3 Hz, H_{glc}-1a), δ 6.19 (d, 1H, J=8.3 Hz, H_{glc}-1b), δ 6.24 (s, 1H, H_J-3), δ 6.47 (s, 1H, H_I-3), δ 6.49 (s, 1H, H_D-3), δ 6.66 (s, 1H, H_E-3), δ 6.98 (s, 2H, H_B-3), δ 7.008 (s, 2H, H_G-3), δ 7.012 (s, 2H, H_C-3), δ 7.02 (s, 2H, H_H-3), δ 7.134 (s, 2H, H_F-3), δ 7.136 (s, 1H, H_A-3).
